# Mendelian randomization study of gastroesophageal reflux disease and major depression

**DOI:** 10.1371/journal.pone.0291086

**Published:** 2023-09-28

**Authors:** Xiaofei Zheng, Xin Zhou, Li Tong, Wang Gu, Siyu Wang, Wenkang Yuang, Chong Zhang, Chaoyang Zhang, Chao Zhang, Bangbei Wan

**Affiliations:** 1 Department of General Surgery, The First Affiliated Hospital of Anhui Medical University, Hefei, China; 2 Qingdao Hospital, University of Health and Rehabilitation Sciences (Qingdao Municipal Hospital), Qingdao, China; 3 Reproductive Medical Center, Hainan Women and Children’s Medical Center, Haikou, China; 4 Department of General Surgery, The Second Affiliated Hospital, School of Medicine, Zhejiang University, Hangzhou, China; Tzu Chi University, TAIWAN

## Abstract

This study systematically investigated the causal relationship between gastroesophageal reflux disease (GERD) and major depression (MD). Single-nucleotide polymorphisms (SNPs) associated with disorders of interest were screened via the genome-wide association study (GWAS) enrolling individuals of European descent. Summary-level data for GERD and MD were extracted from the UK Biobank. The inverse-variance-weighted (IVW) method was utilized as the primary analysis. Sensitivity analyses were performed using the MR-Egger method, the Maximum likelihood method, the MR-pleiotropy residual sum outlier (MR-PRESSO) method, and MR-robust adjusted profile score (MR-RAPS) method. MR-Egger regression, heterogeneity tests, pleiotropy tests, and leave-one-out tests were also performed to analyze sensitivity. The MR Steiger test was used to verify the directionality of the exposure to the outcome. An available website tool (https://shiny.cnsgenomics.com/mRnd/) was used to calculate the statistical power of MR analysis. Meta-analysis was applied to test MD’s average genetically predicted effect on GERD. Our MR study showed a bidirectional causal association between MD and GERD. Regarding MD to GERD, there was a positive association between them; the ORs were 1.500 (95% CI = 1.320–1.704; *P* = 4.91E-10) and 2.058 (95% CI = 1.868–2.267; *P* = 2.20E-48) in the IVW method, respectively. In addition, the meta-analysis also showed a strong positive causal association between MD and GERD. When exposure and outcome were reversed, genetic predisposition to GERD was significantly associated with the overall Risk of advanced MD (ieu-a-1187, OR = 1.982, 95% CI = 1.694–2.319, *P* = 1.41E-17; ieu-b-102, OR = 1.612, 95% CI = 1.530–2.700, *P* = 1.15E-70). Our study provides 100% power to detect the causal effect of MD on GERD and vice versa. Genetically predicted MD was positively associated with higher GERD risk, and vice versa. Our study reminds clinicians to pay attention to screening for GERD when diagnosing and treating MD and vice versa. Moreover, there may be positive feedback between MD and GERD when treating and preventing one disorder may benefit the treatment and prevention of the other.

## Introduction

Gastroesophageal reflux disease (GERD) is a clinically common gastrointestinal disease for all age groups and sexes, with reported prevalence values ranging from 2.5% in China to 51.2% in Greece [1]. Still, the prevalence of GERD symptoms is similar across ethnic groups, and complications of GERD, such as erosive esophagitis and esophageal adenocarcinoma, are more common in white people, especially with central obesity [2,3]. Furthermore, the reported social cost of GERD ranges from $ 12.3 million in Japan to $ 38.9 million in Canada annually [1]. Many risk factors are associated with GERD, including hiatal hernia, obesity, obstructive sleep apnea, and helicobacter pylori gastritis [4–9]. Major GERD phenotypes are non-erosive reflux disease, GERD hypersensitivity, low- or high-grade esophagitis, Barrett’s esophagus, reflux chest pain, laryngopharyngeal reflux, and regurgitation dominant reflux [1]. Many potential complications of GERD, including esophageal adenocarcinoma, bleeding, esophageal rupture, and lung transplant rejection, can lead to death [1]. Necessarily, further exploration of risk factors, which are easily controlled and prevented, for GERD can help design and implement effective prevention strategies or novel treatments.

Major depression (MD) is one of the most common mental disorders worldwide, characterized by persistent low mood [10]. The estimated proportion of the world’s population with MD is 4.4%, making it the leading cause of disability and morbidity worldwide [11–13]. In addition, MD also imposes a substantial economic burden on the United States [14]. Many observational studies have explored the relationship between GERD and MD [15–17]. Accordingly, GERD may play an essential role in the development of MD. Avidan et al. reported that GERD could increase the risk of MD [18]. The National Sample cohort study with 9503 patients said that GERD might act as a risk factor for depressive disorder [19]. Another nationwide cohort study also suggested that GERD may be a risk factor for depressive disorder [20]. According to a systematic review, antidepressants can improve GERD patients’ symptoms [21]. However, the research team of Haug found no significant relationship between GERD and depression. Furthermore, Kessing et al. reported that GERD patients were not with increased depressive levels [22]. Overall, the causal association between MD and GERD is still not fully understood.

Mendelian randomization (MR) has been used as a novel method to assess the potential causal association between multiple diseases [23]. Moreover, MR treats genetic variation as a natural experiment in which individuals are assigned to higher vs lower mean levels of non-genetic exposure. Genetic variants are randomly allocated before birth, never change after birth, and are unaffected by environmental factors. In addition, an MR study can make up for the lack of observation of the survey with residual confounding and reverse causation. The genome-wide association study datasets provide a rich data source for MR studies and are free of charge [24]. Here, we explored the causal association between GERD and MD using the MR method.

## Methods

### Study design

The MR study extracted the summary-level data from the IEU Open GWAS database (https://gwas.mrcieu.ac.uk/). The detailed information on the data used in our research is summarized in S1 Table. The GERD data included in our study is based on self-reporting, where individuals were asked if they have ever been diagnosed by a doctor with heartburn, acid reflux, or acid reflux disease or if they have received treatment for acid reflux/heartburn with medications [25].

### Assumption of the MR study

**Fig 1:**
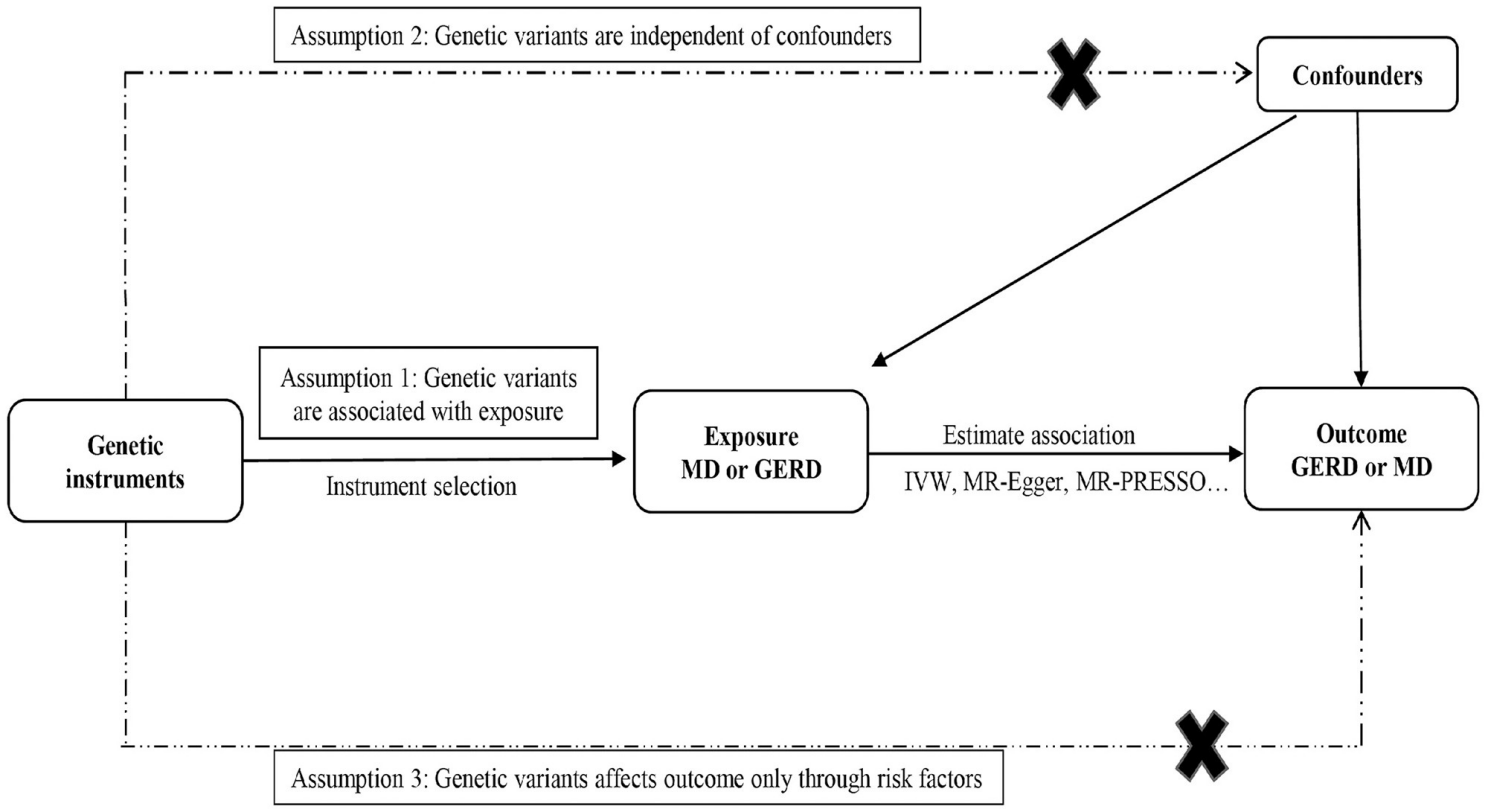
Mendelian Randomization (MR) model. Instrumental variable assumptions: (1) Genetic instrument variables (GIVs) are significantly associated with exposures (*P* < 5 × 10−^8^); (2) GIVs are independent of any possible confounding factors which may influence the relationship between exposure and outcome; (3) The affection of GIVs on the outcome only through exposure without horizontal pleiotropy. MD, major depression; GERD, gastroesophageal reflux disease; IVW, inverse-variance-weighted; MR-PRESSO, MR-pleiotropy residual sum outlier; MR-RAPS, MR-robust adjusted profile score.

The MR research is based on three fundamental assumptions:(1) Genetic instrument variables (GIVs) are significantly associated with exposures. To ensure the establishment of the above assumption, we just investigated two aspects, including the *P* value (P < 5 × 10−^8^) of the GIVs and the clumping method to exclude linkage disequilibrium (LD); (2) GIVs are independent of any possible confounding factors which may influence the relationship between exposure and outcome; (3) The affection of GIVs on the outcome only through exposure without horizontal pleiotropy [26,27] (Fig 1).

### Bidirectional two-sample MR

#### SNP selection

We employed several quality control tests to select SNPs: (1) The SNPs associated with exposures were genome-wide significant (*P* < 5 × 10^−8^); (2) None of the SNPs for the exposure were in linkage. SNPs with a lower *P* value (*P* < 5 × 10^−8^), longer physical distance (≥ 10,000 kb), and a lower likelihood of LD (*r*^*2*^< 0.001) were maintained; (3) The F statistic calculated according to the previous studies for the instrument-exposure correlation was significantly higher than 10 [28,29].

#### Primary analyses

A bidirectional univariable two-sample MR analysis was employed to analyze the association between MD and GERD. The affection of each exposure on outcome was estimated by the inverse-variance-weighted (IVW) model [30].

#### Sensitivity analyses

Besides IVW as the primary method, we also performed MR-Egger [31], maximum likelihood [32], MR-pleiotropy residual sum outlier (MR-PRESSO) [33], and MR-robust adjusted profile score (MR-RAPS) [34] to evaluate the reliability and stability of the results. Assuming that each SNP has the same effect on the outcome, the maximum likelihood method can provide more robust results when measurement errors are present. The MR-Egger and IVW methods were used to assess the heterogeneity, where the P value greater than 0.05 indicated no heterogeneity. The MR-PRESSO, MR-Egger, and IVW methods were performed to identify and remove outliers. The intercept of the MR-Egger model was employed to test the pleiotropy, where a deviation from 0 donates the presence of direction pleiotropy. The leave-one-out method was used to assess the influence of a single SNP on the total effect of IVW. An available website tool (https://shiny.cnsgenomics.com/mRnd/) was used to calculate the statistical power of MR analysis, where a power greater than 80% was considered an excellent value [35]. When the *P* value of the MR Steiget test was lower than 0.05, the causal direction of exposure-causing the outcome was statistically significant [36].

All statistical analyses were implemented via the TwoSampleMR (V 0.5.6) [37] and MRPRESSO packages [33] in R software (V 4.1.2).

### Meat-analysis

In the study, we performed a meta-analysis with two MD-related GWAS datasets to further genetically predict MD’s effect on GERD. The meta-analysis was performed via the "meta" package in R software (V 4.1.2) [38]. Forrest plots were established to assess the consequences of pooling visually. The I2 and the chip-squared-based Q were used to estimate the degree of heterogeneity across studies.

## Results

### Major depressive disorder to gastroesophageal reflux disease

There were two different MD-related GWAS datasets, which can improve the stability and credibility of our MR study. 24 and 37 independent SNPs associated with two other MD datasets were available in the summary statistics for GERD, respectively. The F statistics of all SNPs were greater than 10, suggesting no potential weak instrument bias. The statistical power for the outcome of GERD was approximately equal to 100% (Table 1). The factual information on SNPs used as GIVs is listed in S2 Table.

**Table 1:** MR results of exposure to outcome.

Exposure	outcome	Method	SNPs	OR (95% CI)	*P*	*P*_het	*P*_intercept	*P_*Steiger	F_ Statistic	Power
**MD (ieu-a-1187)**	**GERD**	MR-Egger	24	1.960 (0.790–4.864)	1.61E-01	6.68E-10	5.66E-01	9.7292E-38	177.112	1.000
		**IVW**	**24**	**1.500 (1.320–1.704)**	**4.91E-10**	8.12E-10				
		Maximum likelihood	24	1.516 (1.412–1.629)	2.55E-30					
		MR-PRESSO(RAW)	24	1.500 (1.267–1.775)	2.39E-06					
		MR-RAPS	24	1.555 (1.448–1.669)	0.00E+00					
**MD (ieu-b-102)**	**GERD**	MR-Egger	37	1.902 (1.087–3.331)	3.08E-02	5.68E-09	7.82E-01	3.0531E-25	119.885	1.000
		**IVW**	**37**	**2.058 (1.868–2.267)**	**2.20E-48**	9.28E-09				
		Maximum likelihood	37	2.080 (1.943–2.228)	1.88E-97					
		MR-PRESSO(RAW)	37	2.058 (1.735–2.440)	1.03E-16					
		MR-RAPS	37	2.135 (1.991–2.288)	0.00E+00					
**GERD**	**MDD (ieu-a-1187)**	MR-Egger	8	0.538 (0.134–2.154)	4.15E-01	1.85E-01	1.14E-01	7.66E-03	605.911	1.000
		**IVW**	**8**	**1.982 (1.694–2.319)**	**1.41E-17**	5.41E-02				
		Maximum likelihood	8	2.042 (1.774–2.350)	2.76E-23					
		MR-PRESSO(RAW)	8	1.982 (1.419–2.769)	6.02E-05					
		MR-RAPS	8	2.027 (1.762–2.331)	0.00E+00					
**GERD**	**MDD (ieu-b-102)**	MR-Egger	72	1.394 (1.038–1.872)	3.06E-02	5.04E-11	3.29E-01	8.42E-37	66.675	1.000
		**IVW**	**72**	**1.612 (1.530–1.700)**	**1.15E-70**	3.83E-11				
		Maximum likelihood	72	1.632 (1.570–1.696)	4.12E-135					
		MR-PRESSO(RAW)	72	1.612 (1.480–1.757)	7.56E-28					
		MR-RAPS	72	1.653 (1.590–1.718)	0.00E+00					

MR, Mendelian randomization; MD, major depression; GERD, gastroesophageal reflux disease; SNPs, single nucleotide polymorphisms; OR, odds ratio; GWAS, genome-wide association studies; IVW, inverse-variance-weighted; MR-PRESSO, MR-pleiotropy residual sum outlier; MR-RAPS, MR-robust adjusted profile score.

**Fig 2:**
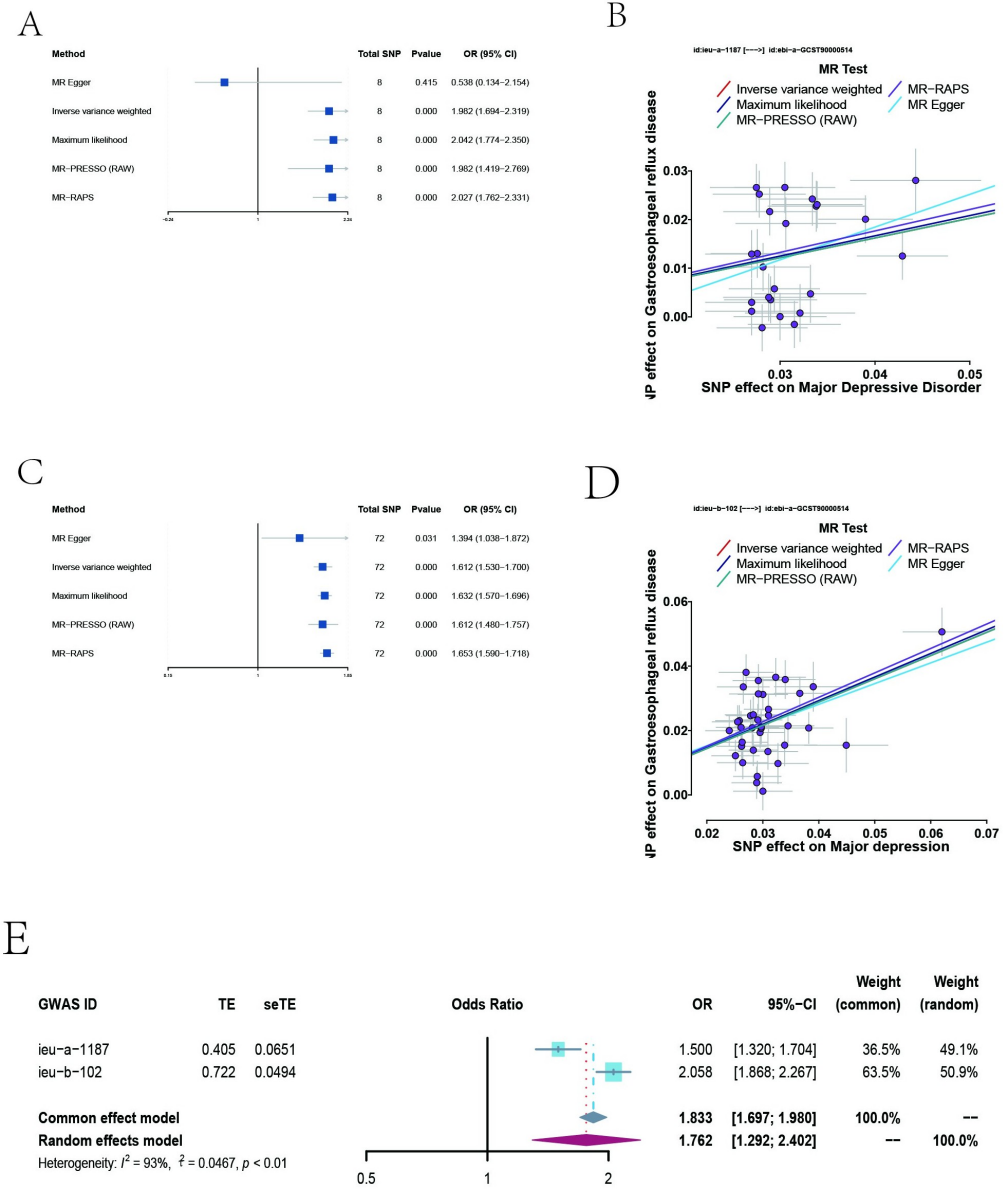
Plots of MR estimates of the causal association between MD and GERD. **A** Forest plot to visualize causal effects of variation in MD (ieu-a-1187) on GERD. Presented OR and CI correspond to the impact of MD on GERD. The results of MR analyses using different analysis methods (MR‒Egger, Maximum likelihood, MR-PRESSO, MR‒RAPS, IVW) are compared. Total single-nucleotide polymorphism (SNP) indicates the number of genetic variants used as instruments for MR analysis. **B**, Scatter plots of MD (ieu-a-1187) with the Risk of GERD. Scatter plot demonstrating the effect of each MD-associated SNP on GERD on the log-odds scale. The slopes of each line represent the causal association for each method. **C**, Forest plot to visualize causal effects of variation in MD (ieu-b-102) on GERD. **D**, Scatter plots of MD (ieu-b-102) with the Risk of GERD. **E**, Forest plots of meta-analysis including two different MD datasets. Forest plots demonstrating the average genetically predicted effect of MD on GERD. Presented OR and CI correspond to the average impact of MD on GERD. *I*^*2*^ statistic and chi-squared-based Q were utilized to assess the heterogeneity across studies. MR, Mendelian randomization; MD, major depression; GERD, gastroesophageal reflux disease; SNPs, single nucleotide polymorphisms; OR, odds ratio; confidence intervals (CI); GWAS, genome-wide association studies; IVW, inverse-variance-weighted; MR-PRESSO, MR-pleiotropy residual sum outlier; MR-RAPS, MR-robust adjusted profile score.

Regarding MD to GERD, there was a positive association between them; the OR was 1.500 (95% CI = 1.320–1.704; *P* = 4.91E-10) and 2.058 (95% CI = 1.868–2.267; *P* = 2.20E-48) in the IVW method, respectively (Table 1, Fig 2A–2D). MR-Egger, Maximum likelihood, MR-PRESSO and MR-RAPS methods showed the same sensitivity, which indicated the results were reliable and stable (Table 1, Fig 2A–2D). Moreover, the density plots revealed that the estimated effect values of most SNPs are distributed within a particular area, indicating no significant heterogeneity in our analysis (S1A and S1B Fig). The intercept term estimated using the MR-Egger method were all centred at the origin (all *P* > 0.05), indicating that the results were not affected by directional pleiotropy (Table 1). Meanwhile, the MR-PRESSO study found no outlier SNPs that increased MR pleiotropy. In addition, no single SNP affected the overall estimate, as demonstrated by the leave-one-out analysis (S3 Table, S1C and S1D Fig). The causal assumption of MD-GERD was verified via the MR Steiger test, which illustrated that the influence of MD on GERD was the correct causal direction (all *P* < 0.01) (Table 1).

### Meta-analysis of Major depressive disorder to gastroesophageal reflux disease

Two different MD-related GWAS datasets were included in the meta-analysis. Heterogeneity between the two datasets was evaluated using *I*^*2*^ tests. The results showed heterogeneity among the two datasets (*I*^*2*^ = 93%, *P* < 0.01), so we used the random effects model to conduct the meta-analysis. The results found a positive effect of MD on the odds of GERD (OR = 1.762, 95% CI = 1.292–2.402, *P* < 0.01), which further emphasized the impact of MD on GERD (Fig 2E).

### Gastroesophageal reflux disease to major depressive disorder

When MD was the outcome, 8 and 72 independent SNPs associated with GERD were available in the summary statistics for two different MD datasets. The F statistics of all SNPs were greater than 10, suggesting no potential weak instrument bias. The statistical power for outcome MD was approximately equal to 100% (Table 1). The factual information on SNPs used as GIVs is listed in S2 Table.

**Fig 3:**
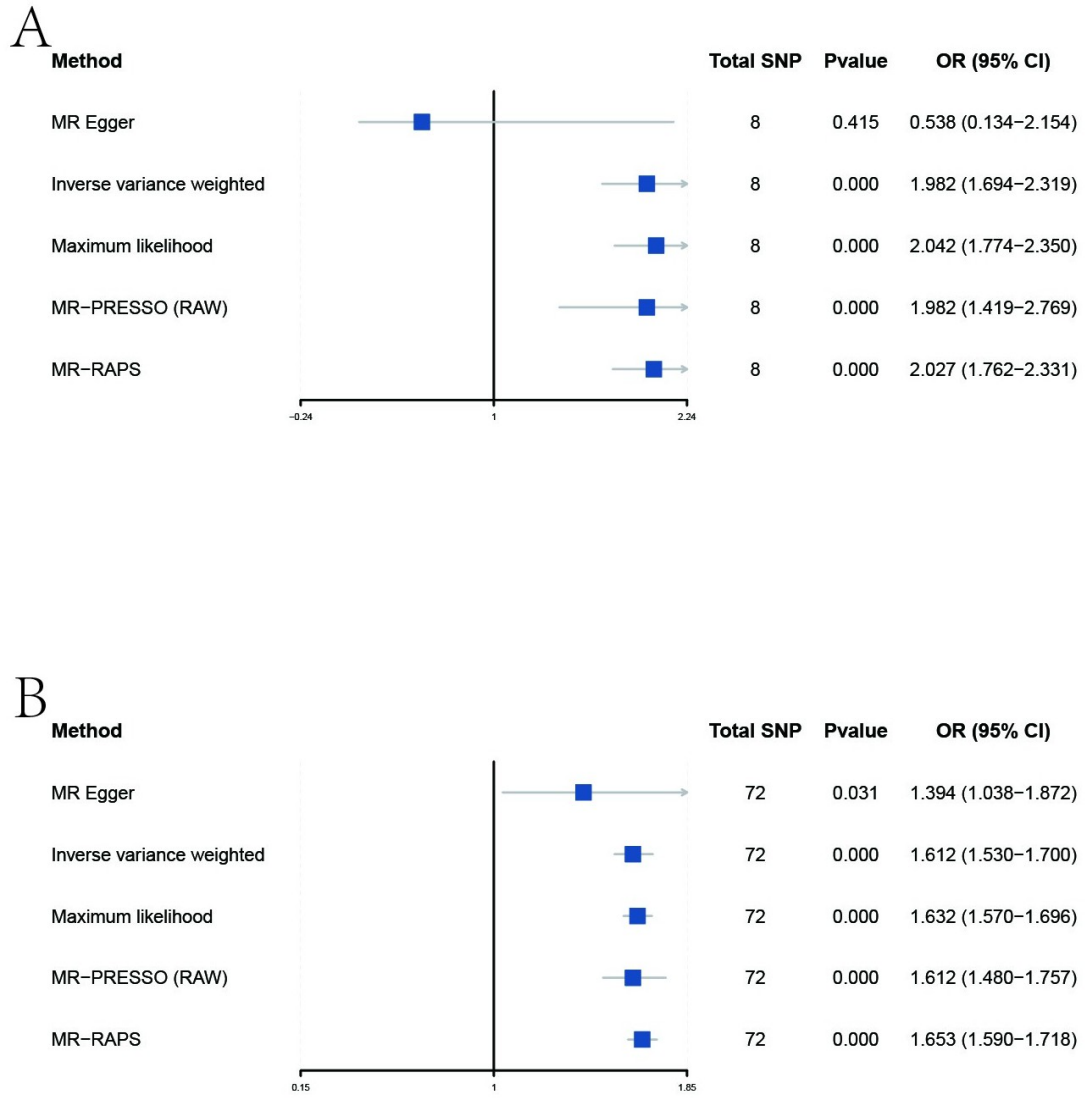
Forest plot to visualize causal effects of variation in GERD on MD. Presented OR and CI correspond to the impact of GERD on MD. The results of MR analyses using different analysis methods (MR‒Egger, Maximum likelihood, MR-PRESSO, MR‒RAPS, IVW) are compared. Total SNP indicates the number of genetic variants used as instruments for MR analysis. MR, Mendelian randomization; MD, major depression; GERD, gastroesophageal reflux disease; SNP, single nucleotide polymorphisms; OR, odds ratio; confidence intervals (CI); IVW, inverse-variance-weighted; MR-PRESSO, MR-pleiotropy residual sum outlier; MR-RAPS, MR-robust adjusted profile score.

Genetic predisposition to GERD was significantly associated with the overall Risk of advanced MD under the IVW method (Table 1, Fig 3A and 3B, S2A and S2D Fig). The results were supported by the other MR method, including Maximum likelihood, MR-PRESSO, and MR-RAPS methods. As shown in S2B and S2E Fig, the estimated effect values of most SNPs are distributed within a particular area, indicating no significant heterogeneity in our analysis. The MR-Egger intercepts revealed that directional pleiotropy did not influence the results (all *P* > 0.05, Table 1). Across the MR-PRESSO analysis, the results found no outlier SNPs that increased MR pleiotropy. Across the leave-one-out analysis, no single SNP affected the overall estimate (S3 Table, S2C and S2F Fig). Under the MR Steiger test, the casual assumption of GERD-MD was the correct direction (all *P* < 0.01) (Table 1).

## Discussion

Our MR results added genetic data to the current discussion about whether MD causes GERD and vice versa. Despite the concern, this discussion leaves open a question that previous traditional observational studies have not yet been able to illustrate. We were the first to investigate the bidirectional causal relationship between MD and GERD under the MR method. We observed that genetically predicated MD were positively associated with GERD and vice versa. Therefore, we hypothesize the presence of a positive feedback loop between MD and GERD, whereby the treatment and prevention of one disorder may be beneficial for treating and preventing the other disorder. Nevertheless, the clinical significance of this outcome remains contingent upon its validation through meticulously conducted clinical trials involving an extensive sample size.

Previous observation epidemiological studies and meta-analyses have explored the associations between major depressive disorder and gastroesophageal reflux disease. In the Korean population, Choi et al. reported a positive association between depression and GERD, especially in the NERD group [17]. According to Yang and his colleagues, depression may increase the risk of GERD and decrease the quality of their life [15]. According to a comparative study, a depression diagnosis played an essential role in developing GERD [16]. In compliance with the above studies, another two cohort studies reported that the depression level was higher in GERD patients than healthy subjects [39,40]. In addition, other studies found that depression was associated with gastrointestinal symptoms [41–43].

Meanwhile, the research team of Avidan found that reflux symptoms were associated with depression disorder [18]. The National Sample cohort study with 9503 patients reported that GERD might act as a risk factor for depressive disorder [19]. Another nationwide cohort study also suggested that GERD may be a risk factor for depressive disorder [20]. In addition, the symptoms of GERD also increase the risk of psychological disorders, including depression [44]. The meta-analysis also demonstrated the relationship between depression and gastroesophageal reflux disease [45,46]. Otherwise, These findings imply that due consideration should be given to the preventive measures and diagnostic approaches for the other ailment in the presence of either MD or GERD.

In conclusion, the above observational and meta-analysis study revealed that GERD could increase the risk of depression. In turn, depression also can increase the risk of GERD. However, potential residual confounders and reverse causality issues may confound the association observed in formal observation studies and meta-analyses. So, our MR study can partially address the limitations of the results of the above studies, and our results revealed a bidirectional causal association between MD and GERD.

Visceral hypersensitivity, characterized by the heightened perception of gastrointestinal stimuli, is a pivotal pathophysiological factor in all functional gastrointestinal disorders, involving sensitization of afferent nerves, spinal dorsal neurons, and alterations in psychoneuroimmunity interactions [47, 48]. Low-dose antidepressants are purported to mitigate visceral nociception while concurrently addressing concomitant anxiety or depressive symptoms. According to some systematic reviews, antidepressants can improve GERD patients’ symptoms [21,49]. Nevertheless, the clinical significance of this outcome remains contingent upon its validation through meticulously conducted clinical trials involving an extensive sample size.

There are several possible explanations for a bidirectional causal association. Most chronic GERD patients have poor sleep quality and cannot rest well, increasing the risk of MD [50]. Next, the abnormal expression of inflammatory cytokines in the esophageal mucosa, including IL-6, IL-8, and tumor necrosis factor-alpha, may sensitize nerve endings in the submucosa of the esophagus by destroying the mucosal barrier [46]. The peripheral inflammation will affect the central nervous system’s inflammation, leading to a psychological disorder, including depression [51–53]. Meanwhile, the psychological disorder may destroy the tight junctions of the esophageal epithelium, further damaging the esophageal mucosa’s barrier function [54]. Psychological disorders, including depression, may ruin the esophageal motor function by decreasing the pressure of the lower esophageal sphincter [55]. Central and peripheral sensitization and the brain-gut axis may also play an essential role in increasing the risk of GERD [54,56–58]. Moreover, depression may promote inflammation and aggravate the condition of GERD, which can be illustrated by an animal experiment [59].

Our MR study has several strengths. We used different methods to evaluate these relationships’ accuracy, and our MR study datasets were the latest. The *P*-value (*P* < 5 × 10^−8^) and LD analyses were applied to assess the relevance assumption. We also used the F statistic to evaluate whether there were weak instrumental variables. In addition, the statistical power for outcome was approximately equal to 100%, which emphasized the strength of the causal effect of exposure on outcome. Because of the nature of the exclusion restriction assumption, many sensitivity analyses were performed to avoid horizontal pleiotropy in our study. The exposure and outcome datasets used in this MR study were all from European populations, which can avoid the confounding effects of diverse people on causal analysis [60].

Moreover, we used two different MD-related GWAS datasets to assess the causal association between major depression and GERD, which can improve the stability and credibility of our MR study. We also perform a meta-analysis to illustrate the causal relationship further. Therefore, we may offer new perspectives that there may be positive feedback between MD and GERD when treating and preventing one disorder may benefit the treatment and prevention of the other.

Admittedly, there are still some limitations in our MR research. The populations of our study were of European ancestry, so the results may not be generalized to persons of other origins. Second, additional factors may confound the results, which cannot be avoided. Thirdly, there is heterogeneity in the instrumental variables, which may be caused by true causality rather than violation of the instrument variable assumption. As for heterogeneity, we used the IVW as the primary analysis method. Fourth, the diagnosis of GERD in our study without diagnostic testing such as endoscopy, reflux monitoring and motility testing. But, MacGregor et al. have previously shown extreme genetic similarity between those broad definitions and clinically diagnosed GERD [61]. So the GERD data included in our study didn’t influence the result of our study.

## Conclusions

In conclusion, our comprehensive MR analysis showed GERD was associated with the Risk for MD, and vice versa, which was critical for better prevention and treatment of MD and GERD. Our study reminds clinicians to pay attention to screening for GERD when diagnosing and treating MD and vice versa. Moreover, there may be positive feedback between MD and GERD when treating and preventing one disorder may benefit the treatment and prevention of the other.

## Supporting information

S1 Fig Density plot and Leave-one-out plots of the MR results of MD to GERD. **A**, **B**, Density plot of the MR results of MD (ieu-a-1187) and MD (ieu-B-102) to GERD. Represent the results of heterogeneity analysis from MD. **C**, **D**, Leave-one-out plots of the MR results of MD (ieu-a-1187) and MD (ieu-b-102). Leave-one-out analysis for IVW MR of MD on GERD in summary-level analyses. MR, Mendelian randomization; MD, major depression; GERD, gastroesophageal reflux disease; SNP, single nucleotide polymorphisms.
(TIF)

S2 Fig Plots of MR estimates of the causal association between GERD and MD. **A**, **D**, Scatter plots of GERD with the Risk of MD (ieu-a-1187) and MD (ieu-b-102). Scatter plot demonstrating the effect of each GERD-associated SNP on MD on the log-odds scale. The slopes of each line represent the causal association for each method. **B**, **E**, Density plot of the MR results of GERD to MD (ieu-a-1187) and MD (ieu-B-102). Represent the results of heterogeneity analysis from GERD. **C**, **F**, Leave-one-out plots of the MR results of GERD. Leave-one-out analysis for IVW MR of GERD on MD (ieu-a-1187) and MD (ieu-B-102) in summary-level analyses. MR, Mendelian randomization; MD, major depression; GERD, gastroesophageal reflux disease; SNP, single nucleotide polymorphisms; IVW, inverse-variance-weighted; MR-PRESSO, MR-pleiotropy residual sum outlier; MR-RAPS, MR-robust adjusted profile score.
(TIF)

S1 Table Characteristics of the GWAS summary data. MD, major depression; GERD, gastroesophageal reflux disease; SNPs, single nucleotide polymorphisms; GWAS, genome-wide association studies.
(DOCX)

S2 Table SNPs are strongly associated with risk factors and their F statistic. SNPs, single nucleotide polymorphisms; EAF, Effect allele frequency.
(DOCX)

S3 Table Leave-one-out results of exposure with the risk of the outcome. SNPs, single nucleotide polymorphisms.
(DOCX)

S1 File. Raw data (XLSX)
