# Supplementary figures and images for: Mendelian randomization study of gastroesophageal reflux disease and major depression
Source: PLoS One. 2023 Sep 28;18(9):e0291086. doi: 10.1371/journal.pone.0291086 (PMC10538746; doi:10.1371/journal.pone.0291086)

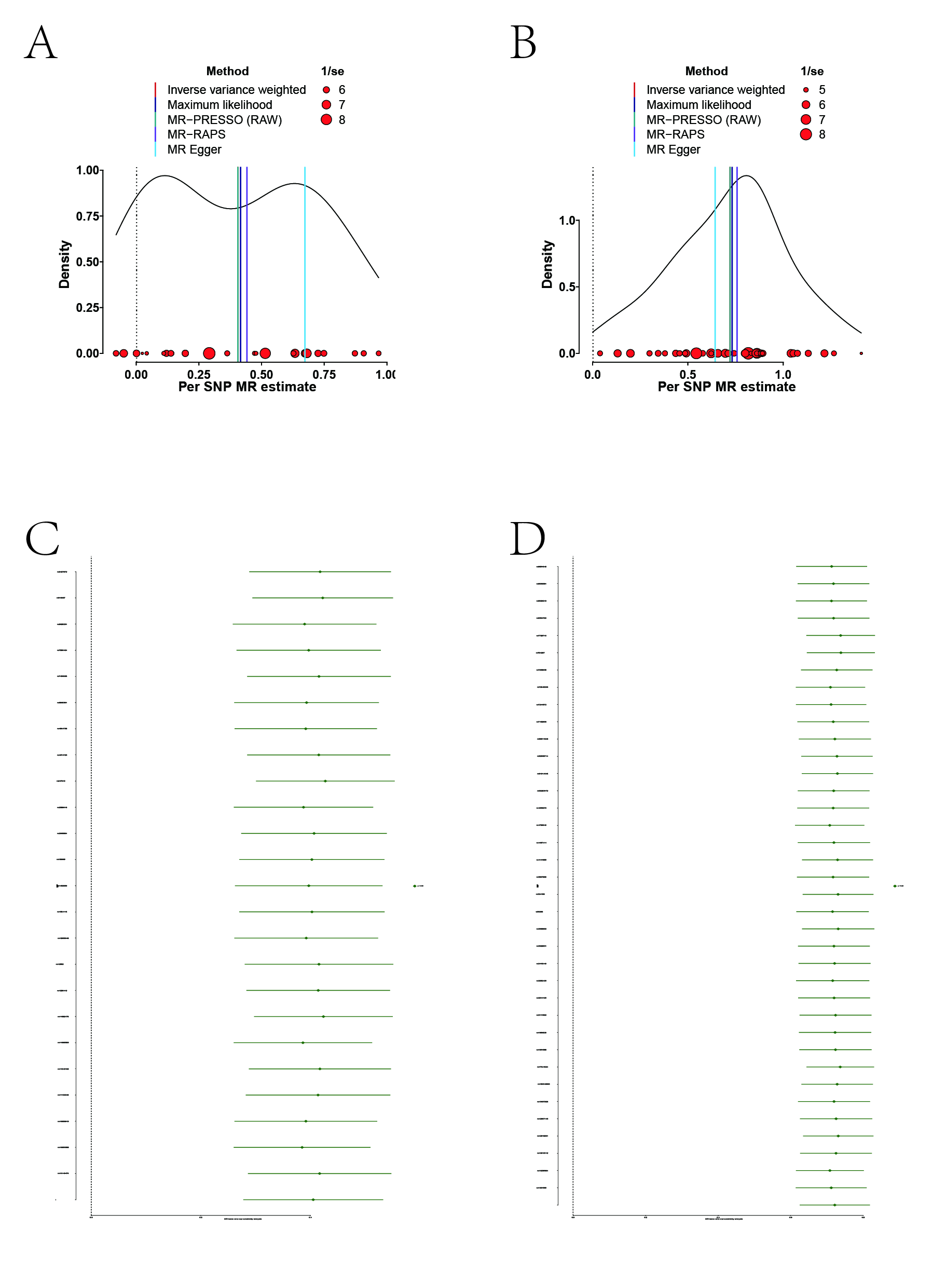

Supplement: S1 Fig — A, B, Density plot of the MR results of MD (ieu-a-1187) and MD (ieu-B-102) to GERD. Represent the results of heterogeneity analysis from MD. C, D, Leave-one-out plots of the MR results of MD (ieu-a-1187) and MD (ieu-b-102). Leave-one-out analysis for IVW MR of MD on GERD in summary-level analyses. MR, Mendelian randomization; MD, major depression; GERD, gastroesophageal reflux disease; SNP, single nucleotide polymorphisms. (TIF) [file pone.0291086.s001.tif]

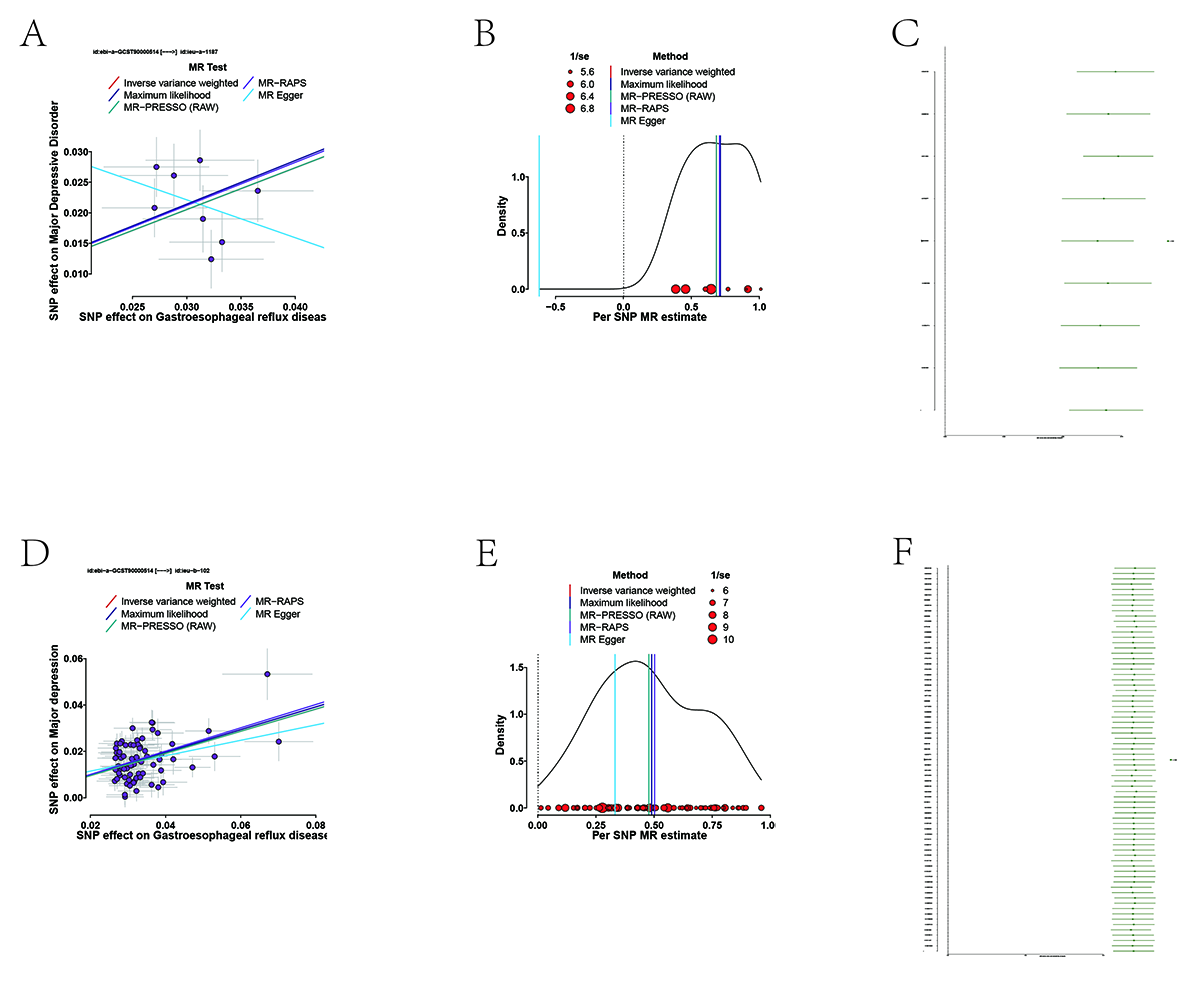

Supplement: S2 Fig — A, D, Scatter plots of GERD with the Risk of MD (ieu-a-1187) and MD (ieu-b-102). Scatter plot demonstrating the effect of each GERD-associated SNP on MD on the log-odds scale. The slopes of each line represent the causal association for each method. B, E, Density plot of the MR results of GERD to MD (ieu-a-1187) and MD (ieu-B-102). Represent the results of heterogeneity analysis from GERD. C, F, Leave-one-out plots of the MR results of GERD. Leave-one-out analysis for IVW MR of GERD on MD (ieu-a-1187) and MD (ieu-B-102) in summary-level analyses. MR, Mendelian randomization; MD, major depression; GERD, gastroesophageal reflux disease; SNP, single nucleotide polymorphisms; IVW, inverse-variance-weighted; MR-PRESSO, MR-pleiotropy residual sum outlier; MR-RAPS, MR-robust adjusted profile score. (TIF) [file pone.0291086.s002.tif]
