# Supplementary material for: Mendelian randomization study of gastroesophageal reflux disease and major depression
Source: PLoS One. 2023 Sep 28;18(9):e0291086. doi: 10.1371/journal.pone.0291086 (PMC10538746; doi:10.1371/journal.pone.0291086)
Supplement: S1 Table — MD, major depression; GERD, gastroesophageal reflux disease; SNPs, single nucleotide polymorphisms; GWAS, genome-wide association studies. (DOCX) [file pone.0291086.s003.docx]

**Table S1. characteristics of the GWAS summary data**.

| **Exposure** | **Ethnicity** | **GWAS ID** | **Total populations** | **PMID** | **SNPs** |
| --- | --- | --- | --- | --- | --- |
| **MD** | European | **ieu-a-1187** | 480,359 | 29700475 | 10,000 |
| **MD** | European | **ieu-b-102** | 500,199 | 30718901 | 30718901 |
| **GERD** | European | **ebi-a-GCST90000514** | 602,604 | 34187846 | 2,320,781 |

MD, major depression; GERD, gastroesophageal reflux disease; SNPs, single nucleotide polymorphisms; GWAS, genome-wide association studies.
