# Supplementary material for: Mendelian randomization study of gastroesophageal reflux disease and major depression
Source: PLoS One. 2023 Sep 28;18(9):e0291086. doi: 10.1371/journal.pone.0291086 (PMC10538746; doi:10.1371/journal.pone.0291086)
Supplement: S2 Table — SNPs, single nucleotide polymorphisms; EAF, Effect allele frequency. (DOCX) [file pone.0291086.s004.docx]

**Table S2. SNPs strongly associated with risk factors and its F statistic.**

| Exposure | Outcome | SNP | Effect allele | Other allele | EAF | Beta | Beta.se | *P*_value | F_ Statistic |
| --- | --- | --- | --- | --- | --- | --- | --- | --- | --- |
| Major Depressive Disorder \|\| id:ieu-a-1187 | Gastroesophageal reflux disease \|\| id:ebi-a-GCST90000514 | rs10149470 | G | A |  | 0.0289964 | 0.0049 | 3.05401e-09 | 35.0183762165764 |
| Major Depressive Disorder \|\| id:ieu-a-1187 | Gastroesophageal reflux disease \|\| id:ebi-a-GCST90000514 | rs10950398 | A | G |  | 0.0274984 | 0.0049 | 2.548e-08 | 31.4936277617659 |
| Major Depressive Disorder \|\| id:ieu-a-1187 | Gastroesophageal reflux disease \|\| id:ebi-a-GCST90000514 | rs10959913 | G | T |  | -0.0333961 | 0.0057 | 5.06198e-09 | 34.3274698433364 |
| Major Depressive Disorder \|\| id:ieu-a-1187 | Gastroesophageal reflux disease \|\| id:ebi-a-GCST90000514 | rs11135349 | C | A |  | 0.0293979 | 0.0048 | 1.09199e-09 | 37.5102658164063 |
| Major Depressive Disorder \|\| id:ieu-a-1187 | Gastroesophageal reflux disease \|\| id:ebi-a-GCST90000514 | rs11643192 | A | C |  | 0.0270021 | 0.0049 | 3.359e-08 | 30.3670722369846 |
| Major Depressive Disorder \|\| id:ieu-a-1187 | Gastroesophageal reflux disease \|\| id:ebi-a-GCST90000514 | rs11663393 | A | G |  | 0.0278 | 0.0049 | 1.64502e-08 | 32.1882548937943 |
| Major Depressive Disorder \|\| id:ieu-a-1187 | Gastroesophageal reflux disease \|\| id:ebi-a-GCST90000514 | rs11682175 | C | T |  | 0.0281012 | 0.0048 | 4.68004e-09 | 34.2741945069445 |
| Major Depressive Disorder \|\| id:ieu-a-1187 | Gastroesophageal reflux disease \|\| id:ebi-a-GCST90000514 | rs1226412 | T | C |  | 0.0332026 | 0.0059 | 2.38402e-08 | 31.6694239230106 |
| Major Depressive Disorder \|\| id:ieu-a-1187 | Gastroesophageal reflux disease \|\| id:ebi-a-GCST90000514 | rs12552 | G | A |  | -0.0428966 | 0.0048 | 6.07156e-19 | 79.8662452934028 |
| Major Depressive Disorder \|\| id:ieu-a-1187 | Gastroesophageal reflux disease \|\| id:ebi-a-GCST90000514 | rs12958048 | G | A |  | -0.0338022 | 0.0051 | 3.61327e-11 | 43.9288244844291 |
| Major Depressive Disorder \|\| id:ieu-a-1187 | Gastroesophageal reflux disease \|\| id:ebi-a-GCST90000514 | rs1354115 | A | C |  | 0.0275957 | 0.0049 | 2.37001e-08 | 31.7168953973345 |
| Major Depressive Disorder \|\| id:ieu-a-1187 | Gastroesophageal reflux disease \|\| id:ebi-a-GCST90000514 | rs1432639 | A | C |  | 0.0389995 | 0.005 | 4.55302e-15 | 60.83844001 |
| Major Depressive Disorder \|\| id:ieu-a-1187 | Gastroesophageal reflux disease \|\| id:ebi-a-GCST90000514 | rs159963 | A | C |  | -0.0270013 | 0.0049 | 3.19301e-08 | 30.3652728733861 |
| Major Depressive Disorder \|\| id:ieu-a-1187 | Gastroesophageal reflux disease \|\| id:ebi-a-GCST90000514 | rs2005864 | T | C |  | 0.0281987 | 0.0049 | 6.73101e-09 | 33.1181458429821 |
| Major Depressive Disorder \|\| id:ieu-a-1187 | Gastroesophageal reflux disease \|\| id:ebi-a-GCST90000514 | rs2389016 | T | C |  | 0.0305001 | 0.0053 | 1.01801e-08 | 33.1169846924172 |
| Major Depressive Disorder \|\| id:ieu-a-1187 | Gastroesophageal reflux disease \|\| id:ebi-a-GCST90000514 | rs247910 | G | A |  | 0.031501 | 0.0049 | 1.065e-10 | 41.3291545605998 |
| Major Depressive Disorder \|\| id:ieu-a-1187 | Gastroesophageal reflux disease \|\| id:ebi-a-GCST90000514 | rs4074723 | C | A |  | 0.0270013 | 0.0049 | 3.11803e-08 | 30.3652728733861 |
| Major Depressive Disorder \|\| id:ieu-a-1187 | Gastroesophageal reflux disease \|\| id:ebi-a-GCST90000514 | rs4904738 | C | T |  | 0.0289037 | 0.0049 | 2.57199e-09 | 34.7948302244898 |
| Major Depressive Disorder \|\| id:ieu-a-1187 | Gastroesophageal reflux disease \|\| id:ebi-a-GCST90000514 | rs6905391 | A | G |  | -0.0442968 | 0.0069 | 1.348e-10 | 41.2141669867675 |
| Major Depressive Disorder \|\| id:ieu-a-1187 | Gastroesophageal reflux disease \|\| id:ebi-a-GCST90000514 | rs7430565 | A | G |  | -0.0288008 | 0.0048 | 2.86801e-09 | 36.0020000277778 |
| Major Depressive Disorder \|\| id:ieu-a-1187 | Gastroesophageal reflux disease \|\| id:ebi-a-GCST90000514 | rs7856424 | T | C |  | -0.0306035 | 0.0053 | 8.47891e-09 | 33.3419085884656 |
| Major Depressive Disorder \|\| id:ieu-a-1187 | Gastroesophageal reflux disease \|\| id:ebi-a-GCST90000514 | rs8025231 | C | A |  | 0.0338981 | 0.0048 | 2.35776e-12 | 49.8733152608507 |
| Major Depressive Disorder \|\| id:ieu-a-1187 | Gastroesophageal reflux disease \|\| id:ebi-a-GCST90000514 | rs915057 | G | A |  | 0.0299954 | 0.0049 | 7.60904e-10 | 37.4728871786755 |
| Major Depressive Disorder \|\| id:ieu-a-1187 | Gastroesophageal reflux disease \|\| id:ebi-a-GCST90000514 | rs9427672 | G | A |  | 0.0320997 | 0.0058 | 3.11903e-08 | 30.6299268754459 |
| Major depression \|\| id:ieu-b-102 | Gastroesophageal reflux disease \|\| id:ebi-a-GCST90000514 | rs1021363 | G | A | 0.6434 | -0.03 | 0.0045 | 2.28718e-11 | 44.4444444444444 |
| Major depression \|\| id:ieu-b-102 | Gastroesophageal reflux disease \|\| id:ebi-a-GCST90000514 | rs10235664 | C | T | 0.2529 | -0.027 | 0.0049 | 4.67703e-08 | 30.3623490212411 |
| Major depression \|\| id:ieu-b-102 | Gastroesophageal reflux disease \|\| id:ebi-a-GCST90000514 | rs10913112 | T | C | 0.378 | -0.0262 | 0.0045 | 4.52501e-09 | 33.8982716049383 |
| Major depression \|\| id:ieu-b-102 | Gastroesophageal reflux disease \|\| id:ebi-a-GCST90000514 | rs12919291 | C | G | 0.1884 | 0.0327 | 0.0055 | 3.092e-09 | 35.3484297520661 |
| Major depression \|\| id:ieu-b-102 | Gastroesophageal reflux disease \|\| id:ebi-a-GCST90000514 | rs12967143 | C | G | 0.7012 | -0.0345 | 0.0047 | 2.52697e-13 | 53.8818469895881 |
| Major depression \|\| id:ieu-b-102 | Gastroesophageal reflux disease \|\| id:ebi-a-GCST90000514 | rs13037326 | T | C | 0.2597 | 0.031 | 0.0049 | 2.398e-10 | 40.0249895876718 |
| Major depression \|\| id:ieu-b-102 | Gastroesophageal reflux disease \|\| id:ebi-a-GCST90000514 | rs150346963 | T | C | 0.4118 | 0.0283 | 0.0044 | 1.15699e-10 | 41.3682851239669 |
| Major depression \|\| id:ieu-b-102 | Gastroesophageal reflux disease \|\| id:ebi-a-GCST90000514 | rs17641524 | T | C | 0.2101 | -0.03 | 0.0053 | 1.502e-08 | 32.0398718405126 |
| Major depression \|\| id:ieu-b-102 | Gastroesophageal reflux disease \|\| id:ebi-a-GCST90000514 | rs1931388 | G | A | 0.4042 | -0.0295 | 0.0044 | 1.67996e-11 | 44.9509297520661 |
| Major depression \|\| id:ieu-b-102 | Gastroesophageal reflux disease \|\| id:ebi-a-GCST90000514 | rs1950829 | G | A | 0.5173 | -0.0297 | 0.0043 | 4.73805e-12 | 47.7063277447269 |
| Major depression \|\| id:ieu-b-102 | Gastroesophageal reflux disease \|\| id:ebi-a-GCST90000514 | rs2111592 | A | G | 0.3141 | 0.0263 | 0.0046 | 1.34999e-08 | 32.6885633270321 |
| Major depression \|\| id:ieu-b-102 | Gastroesophageal reflux disease \|\| id:ebi-a-GCST90000514 | rs2214123 | G | A | 0.6466 | -0.0261 | 0.0045 | 8.55598e-09 | 33.64 |
| Major depression \|\| id:ieu-b-102 | Gastroesophageal reflux disease \|\| id:ebi-a-GCST90000514 | rs2232423 | G | A | 0.1056 | -0.062 | 0.007 | 1.13501e-18 | 78.4489795918367 |
| Major depression \|\| id:ieu-b-102 | Gastroesophageal reflux disease \|\| id:ebi-a-GCST90000514 | rs2418449 | C | T | 0.281 | -0.0281 | 0.0048 | 4.24502e-09 | 34.2712673611111 |
| Major depression \|\| id:ieu-b-102 | Gastroesophageal reflux disease \|\| id:ebi-a-GCST90000514 | rs2522831 | C | T | 0.4739 | 0.024 | 0.0043 | 2.113e-08 | 31.1519740400216 |
| Major depression \|\| id:ieu-b-102 | Gastroesophageal reflux disease \|\| id:ebi-a-GCST90000514 | rs2568958 | A | G | 0.6042 | 0.0382 | 0.0044 | 2.90202e-18 | 75.3739669421488 |
| Major depression \|\| id:ieu-b-102 | Gastroesophageal reflux disease \|\| id:ebi-a-GCST90000514 | rs30266 | A | G | 0.3271 | 0.0366 | 0.0046 | 1.42791e-15 | 63.3062381852552 |
| Major depression \|\| id:ieu-b-102 | Gastroesophageal reflux disease \|\| id:ebi-a-GCST90000514 | rs354155 | C | G | 0.0923 | -0.0449 | 0.0075 | 1.75102e-09 | 35.8401777777778 |
| Major depression \|\| id:ieu-b-102 | Gastroesophageal reflux disease \|\| id:ebi-a-GCST90000514 | rs3807865 | A | G | 0.4105 | 0.031 | 0.0044 | 1.09295e-12 | 49.6384297520661 |
| Major depression \|\| id:ieu-b-102 | Gastroesophageal reflux disease \|\| id:ebi-a-GCST90000514 | rs4141983 | C | T | 0.326 | -0.0264 | 0.0046 | 9.69192e-09 | 32.937618147448 |
| Major depression \|\| id:ieu-b-102 | Gastroesophageal reflux disease \|\| id:ebi-a-GCST90000514 | rs4497414 | C | T | 0.44 | 0.0291 | 0.0044 | 2.92685e-11 | 43.7401859504132 |
| Major depression \|\| id:ieu-b-102 | Gastroesophageal reflux disease \|\| id:ebi-a-GCST90000514 | rs4799949 | T | C | 0.6684 | -0.0292 | 0.0046 | 1.40301e-10 | 40.2948960302457 |
| Major depression \|\| id:ieu-b-102 | Gastroesophageal reflux disease \|\| id:ebi-a-GCST90000514 | rs4936276 | C | G | 0.622 | 0.0278 | 0.0044 | 3.57001e-10 | 39.9194214876033 |
| Major depression \|\| id:ieu-b-102 | Gastroesophageal reflux disease \|\| id:ebi-a-GCST90000514 | rs59283172 | A | G | 0.1081 | -0.039 | 0.007 | 2.40902e-08 | 31.0408163265306 |
| Major depression \|\| id:ieu-b-102 | Gastroesophageal reflux disease \|\| id:ebi-a-GCST90000514 | rs61914045 | A | G | 0.2034 | 0.0309 | 0.0054 | 7.96398e-09 | 32.7438271604938 |
| Major depression \|\| id:ieu-b-102 | Gastroesophageal reflux disease \|\| id:ebi-a-GCST90000514 | rs62535714 | A | G | 0.1639 | 0.0339 | 0.0058 | 4.68598e-09 | 34.1620095124851 |
| Major depression \|\| id:ieu-b-102 | Gastroesophageal reflux disease \|\| id:ebi-a-GCST90000514 | rs66511648 | C | T | 0.284 | 0.0297 | 0.0048 | 6.03004e-10 | 38.28515625 |
| Major depression \|\| id:ieu-b-102 | Gastroesophageal reflux disease \|\| id:ebi-a-GCST90000514 | rs7152906 | C | T | 0.5196 | 0.0258 | 0.0043 | 1.87301e-09 | 36 |
| Major depression \|\| id:ieu-b-102 | Gastroesophageal reflux disease \|\| id:ebi-a-GCST90000514 | rs7241572 | A | G | 0.2047 | 0.0323 | 0.0054 | 2.43299e-09 | 35.7781207133059 |
| Major depression \|\| id:ieu-b-102 | Gastroesophageal reflux disease \|\| id:ebi-a-GCST90000514 | rs72948506 | A | G | 0.2975 | 0.0265 | 0.0047 | 1.71498e-08 | 31.7904028972386 |
| Major depression \|\| id:ieu-b-102 | Gastroesophageal reflux disease \|\| id:ebi-a-GCST90000514 | rs7538938 | C | T | 0.5599 | 0.0251 | 0.0043 | 7.28903e-09 | 34.0730124391563 |
| Major depression \|\| id:ieu-b-102 | Gastroesophageal reflux disease \|\| id:ebi-a-GCST90000514 | rs754287 | A | T | 0.3664 | -0.0289 | 0.0045 | 1.31099e-10 | 41.2449382716049 |
| Major depression \|\| id:ieu-b-102 | Gastroesophageal reflux disease \|\| id:ebi-a-GCST90000514 | rs7725715 | A | G | 0.5343 | 0.029 | 0.0043 | 1.60694e-11 | 45.4840454299621 |
| Major depression \|\| id:ieu-b-102 | Gastroesophageal reflux disease \|\| id:ebi-a-GCST90000514 | rs9364755 | G | A | 0.2262 | 0.0283 | 0.0051 | 3.48602e-08 | 30.7916186082276 |
| Major depression \|\| id:ieu-b-102 | Gastroesophageal reflux disease \|\| id:ebi-a-GCST90000514 | rs9529218 | T | C | 0.2031 | -0.034 | 0.0054 | 2.231e-10 | 39.6433470507545 |
| Major depression \|\| id:ieu-b-102 | Gastroesophageal reflux disease \|\| id:ebi-a-GCST90000514 | rs9536381 | T | C | 0.3259 | 0.0255 | 0.0046 | 2.61698e-08 | 30.7301512287335 |
| Major depression \|\| id:ieu-b-102 | Gastroesophageal reflux disease \|\| id:ebi-a-GCST90000514 | rs9831648 | T | G | 0.7739 | -0.0292 | 0.0052 | 1.58599e-08 | 31.5325443786982 |
| Gastroesophageal reflux disease \|\| id:ebi-a-GCST90000514 | Major Depressive Disorder \|\| id:ieu-a-1187 | rs1021363 | G | A | 0.641992 | -0.031217 | 0.00502204 | 5.09976e-10 | 38.6386544156877 |
| Gastroesophageal reflux disease \|\| id:ebi-a-GCST90000514 | Major Depressive Disorder \|\| id:ieu-a-1187 | rs12204714 | T | C | 0.632223 | -0.028817 | 0.00499422 | 7.9241e-09 | 33.2937101262284 |
| Gastroesophageal reflux disease \|\| id:ebi-a-GCST90000514 | Major Depressive Disorder \|\| id:ieu-a-1187 | rs12967855 | G | A | 0.670435 | -0.0365451 | 0.00513354 | 1.08793e-12 | 50.6785766086618 |
| Gastroesophageal reflux disease \|\| id:ebi-a-GCST90000514 | Major Depressive Disorder \|\| id:ieu-a-1187 | rs2043539 | A | G | 0.41866 | 0.0272058 | 0.00486485 | 2.24042e-08 | 31.2740476908994 |
| Gastroesophageal reflux disease \|\| id:ebi-a-GCST90000514 | Major Depressive Disorder \|\| id:ieu-a-1187 | rs3793577 | G | A | 0.538279 | 0.0270309 | 0.00484976 | 2.49436e-08 | 31.0656551755071 |
| Gastroesophageal reflux disease \|\| id:ebi-a-GCST90000514 | Major Depressive Disorder \|\| id:ieu-a-1187 | rs6711584 | A | G | 0.452019 | 0.0322545 | 0.00483974 | 2.65583e-11 | 44.4157055825809 |
| Gastroesophageal reflux disease \|\| id:ebi-a-GCST90000514 | Major Depressive Disorder \|\| id:ieu-a-1187 | rs9396740 | A | G | 0.248794 | -0.031493 | 0.0055587 | 1.46562e-08 | 32.0982676657298 |
| Gastroesophageal reflux disease \|\| id:ebi-a-GCST90000514 | Major Depressive Disorder \|\| id:ieu-a-1187 | rs9940128 | A | G | 0.421755 | 0.0332513 | 0.00486305 | 8.05749e-12 | 46.7519563532776 |
| Gastroesophageal reflux disease \|\| id:ebi-a-GCST90000514 | Major depression \|\| id:ieu-b-102 | rs10010963 | T | C | 0.616433 | -0.0269803 | 0.00494665 | 4.91779e-08 | 29.7489185641032 |
| Gastroesophageal reflux disease \|\| id:ebi-a-GCST90000514 | Major depression \|\| id:ieu-b-102 | rs1011407 | G | A | 0.121628 | -0.0420618 | 0.00735917 | 1.09343e-08 | 32.6676607755557 |
| Gastroesophageal reflux disease \|\| id:ebi-a-GCST90000514 | Major depression \|\| id:ieu-b-102 | rs10133111 | A | G | 0.162996 | 0.0417875 | 0.00650789 | 1.35338e-10 | 41.2299085888202 |
| Gastroesophageal reflux disease \|\| id:ebi-a-GCST90000514 | Major depression \|\| id:ieu-b-102 | rs1021363 | G | A | 0.641992 | -0.031217 | 0.00502204 | 5.09976e-10 | 38.6386544156877 |
| Gastroesophageal reflux disease \|\| id:ebi-a-GCST90000514 | Major depression \|\| id:ieu-b-102 | rs10837002 | G | C | 0.35122 | 0.0276491 | 0.00503665 | 4.02893e-08 | 30.1355036021332 |
| Gastroesophageal reflux disease \|\| id:ebi-a-GCST90000514 | Major depression \|\| id:ieu-b-102 | rs11762636 | A | C | 0.180282 | -0.0514827 | 0.00625598 | 1.88235e-16 | 67.7223355629788 |
| Gastroesophageal reflux disease \|\| id:ebi-a-GCST90000514 | Major depression \|\| id:ieu-b-102 | rs11953061 | T | C | 0.338908 | 0.0281599 | 0.00508694 | 3.09949e-08 | 30.6442492482656 |
| Gastroesophageal reflux disease \|\| id:ebi-a-GCST90000514 | Major depression \|\| id:ieu-b-102 | rs12204714 | T | C | 0.632223 | -0.028817 | 0.00499422 | 7.9241e-09 | 33.2937101262284 |
| Gastroesophageal reflux disease \|\| id:ebi-a-GCST90000514 | Major depression \|\| id:ieu-b-102 | rs12357321 | A | G | 0.311087 | 0.0317159 | 0.00523077 | 1.33325e-09 | 36.764006242612 |
| Gastroesophageal reflux disease \|\| id:ebi-a-GCST90000514 | Major depression \|\| id:ieu-b-102 | rs12453010 | T | C | 0.394803 | 0.0296967 | 0.00493315 | 1.74606e-09 | 36.2382937658438 |
| Gastroesophageal reflux disease \|\| id:ebi-a-GCST90000514 | Major depression \|\| id:ieu-b-102 | rs12598916 | G | C | 0.274798 | -0.0332614 | 0.00539171 | 6.8729e-10 | 38.0564260116615 |
| Gastroesophageal reflux disease \|\| id:ebi-a-GCST90000514 | Major depression \|\| id:ieu-b-102 | rs12967855 | G | A | 0.670435 | -0.0365451 | 0.00513354 | 1.08793e-12 | 50.6785766086618 |
| Gastroesophageal reflux disease \|\| id:ebi-a-GCST90000514 | Major depression \|\| id:ieu-b-102 | rs12997558 | A | G | 0.358751 | 0.0278184 | 0.00502214 | 3.03941e-08 | 30.6822118821974 |
| Gastroesophageal reflux disease \|\| id:ebi-a-GCST90000514 | Major depression \|\| id:ieu-b-102 | rs13107325 | T | C | 0.074445 | 0.0701443 | 0.00918312 | 2.19938e-14 | 58.3450816821714 |
| Gastroesophageal reflux disease \|\| id:ebi-a-GCST90000514 | Major depression \|\| id:ieu-b-102 | rs1334297 | A | G | 0.734249 | -0.0387984 | 0.00545512 | 1.1413e-12 | 50.5846809742321 |
| Gastroesophageal reflux disease \|\| id:ebi-a-GCST90000514 | Major depression \|\| id:ieu-b-102 | rs13409451 | G | A | 0.392403 | -0.0277081 | 0.00493183 | 1.92943e-08 | 31.5643824290726 |
| Gastroesophageal reflux disease \|\| id:ebi-a-GCST90000514 | Major depression \|\| id:ieu-b-102 | rs1431196 | G | A | 0.428432 | 0.0324197 | 0.00486448 | 2.65461e-11 | 44.4165824103886 |
| Gastroesophageal reflux disease \|\| id:ebi-a-GCST90000514 | Major depression \|\| id:ieu-b-102 | rs1479405 | T | C | 0.3217 | 0.0314843 | 0.0051514 | 9.85213e-10 | 37.3540363115271 |
| Gastroesophageal reflux disease \|\| id:ebi-a-GCST90000514 | Major depression \|\| id:ieu-b-102 | rs1510719 | C | T | 0.383439 | -0.0388844 | 0.00494701 | 3.83619e-15 | 61.7824643912677 |
| Gastroesophageal reflux disease \|\| id:ebi-a-GCST90000514 | Major depression \|\| id:ieu-b-102 | rs1596747 | G | A | 0.494136 | 0.0310869 | 0.00480742 | 1.00344e-10 | 41.8148651458329 |
| Gastroesophageal reflux disease \|\| id:ebi-a-GCST90000514 | Major depression \|\| id:ieu-b-102 | rs1716171 | T | C | 0.790024 | 0.0383981 | 0.00590382 | 7.82528e-11 | 42.3012552413647 |
| Gastroesophageal reflux disease \|\| id:ebi-a-GCST90000514 | Major depression \|\| id:ieu-b-102 | rs17379561 | T | A | 0.144391 | 0.0530714 | 0.00686573 | 1.07622e-14 | 59.7513397632777 |
| Gastroesophageal reflux disease \|\| id:ebi-a-GCST90000514 | Major depression \|\| id:ieu-b-102 | rs1883842 | G | T | 0.279255 | 0.0308332 | 0.00536826 | 9.26766e-09 | 32.9890733389932 |
| Gastroesophageal reflux disease \|\| id:ebi-a-GCST90000514 | Major depression \|\| id:ieu-b-102 | rs1937450 | G | T | 0.537739 | 0.0315845 | 0.00484484 | 7.06806e-11 | 42.5000199664504 |
| Gastroesophageal reflux disease \|\| id:ebi-a-GCST90000514 | Major depression \|\| id:ieu-b-102 | rs2016933 | G | C | 0.730053 | -0.0310253 | 0.00542057 | 1.04275e-08 | 32.7598620937356 |
| Gastroesophageal reflux disease \|\| id:ebi-a-GCST90000514 | Major depression \|\| id:ieu-b-102 | rs2023878 | T | C | 0.192377 | -0.0362846 | 0.00611913 | 3.03501e-09 | 35.161332087539 |
| Gastroesophageal reflux disease \|\| id:ebi-a-GCST90000514 | Major depression \|\| id:ieu-b-102 | rs2106353 | T | G | 0.231451 | 0.0367491 | 0.00572505 | 1.37177e-10 | 41.2035700818251 |
| Gastroesophageal reflux disease \|\| id:ebi-a-GCST90000514 | Major depression \|\| id:ieu-b-102 | rs215614 | A | G | 0.629725 | -0.0328541 | 0.00497715 | 4.08413e-11 | 43.573022883328 |
| Gastroesophageal reflux disease \|\| id:ebi-a-GCST90000514 | Major depression \|\| id:ieu-b-102 | rs2164300 | T | C | 0.523279 | -0.0264751 | 0.00482677 | 4.13352e-08 | 30.0858308022694 |
| Gastroesophageal reflux disease \|\| id:ebi-a-GCST90000514 | Major depression \|\| id:ieu-b-102 | rs2240326 | A | G | 0.473775 | -0.0471681 | 0.00481335 | 1.13214e-22 | 96.0288837396784 |
| Gastroesophageal reflux disease \|\| id:ebi-a-GCST90000514 | Major depression \|\| id:ieu-b-102 | rs2396133 | G | A | 0.475329 | 0.0293547 | 0.00481787 | 1.10889e-09 | 37.12317717013 |
| Gastroesophageal reflux disease \|\| id:ebi-a-GCST90000514 | Major depression \|\| id:ieu-b-102 | rs2396766 | A | G | 0.47308 | 0.0322057 | 0.00481878 | 2.33507e-11 | 44.667463159539 |
| Gastroesophageal reflux disease \|\| id:ebi-a-GCST90000514 | Major depression \|\| id:ieu-b-102 | rs2734839 | T | C | 0.606693 | -0.0283478 | 0.00492778 | 8.78537e-09 | 33.0929969254255 |
| Gastroesophageal reflux disease \|\| id:ebi-a-GCST90000514 | Major depression \|\| id:ieu-b-102 | rs2744961 | T | C | 0.358437 | 0.0292007 | 0.00501549 | 5.81126e-09 | 33.8968847925483 |
| Gastroesophageal reflux disease \|\| id:ebi-a-GCST90000514 | Major depression \|\| id:ieu-b-102 | rs2782641 | A | G | 0.612669 | 0.0270882 | 0.00494583 | 4.32643e-08 | 29.9972833575794 |
| Gastroesophageal reflux disease \|\| id:ebi-a-GCST90000514 | Major depression \|\| id:ieu-b-102 | rs2834005 | C | T | 0.315 | 0.0296997 | 0.0051734 | 9.42041e-09 | 32.9573290051133 |
| Gastroesophageal reflux disease \|\| id:ebi-a-GCST90000514 | Major depression \|\| id:ieu-b-102 | rs2838771 | C | G | 0.646721 | -0.0280984 | 0.00506571 | 2.90964e-08 | 30.7668145457597 |
| Gastroesophageal reflux disease \|\| id:ebi-a-GCST90000514 | Major depression \|\| id:ieu-b-102 | rs324769 | T | C | 0.449179 | -0.0267699 | 0.00483328 | 3.04775e-08 | 30.676766960777 |
| Gastroesophageal reflux disease \|\| id:ebi-a-GCST90000514 | Major depression \|\| id:ieu-b-102 | rs329122 | A | G | 0.419631 | -0.0289529 | 0.00488358 | 3.05485e-09 | 35.1485592395076 |
| Gastroesophageal reflux disease \|\| id:ebi-a-GCST90000514 | Major depression \|\| id:ieu-b-102 | rs3766823 | A | G | 0.171467 | 0.0393599 | 0.00638545 | 7.09316e-10 | 37.9948693571839 |
| Gastroesophageal reflux disease \|\| id:ebi-a-GCST90000514 | Major depression \|\| id:ieu-b-102 | rs3793577 | G | A | 0.538279 | 0.0270309 | 0.00484976 | 2.49436e-08 | 31.0656551755071 |
| Gastroesophageal reflux disease \|\| id:ebi-a-GCST90000514 | Major depression \|\| id:ieu-b-102 | rs3828917 | T | G | 0.041826 | 0.0671113 | 0.0120054 | 2.26945e-08 | 31.2491374184425 |
| Gastroesophageal reflux disease \|\| id:ebi-a-GCST90000514 | Major depression \|\| id:ieu-b-102 | rs3863241 | T | C | 0.52696 | 0.0324982 | 0.00481521 | 1.48799e-11 | 45.5499757061267 |
| Gastroesophageal reflux disease \|\| id:ebi-a-GCST90000514 | Major depression \|\| id:ieu-b-102 | rs4300861 | T | C | 0.38208 | 0.0307132 | 0.00494886 | 5.431e-10 | 38.5158777423106 |
| Gastroesophageal reflux disease \|\| id:ebi-a-GCST90000514 | Major depression \|\| id:ieu-b-102 | rs4382592 | G | T | 0.699524 | -0.0302679 | 0.00525091 | 8.19917e-09 | 33.2273290073754 |
| Gastroesophageal reflux disease \|\| id:ebi-a-GCST90000514 | Major depression \|\| id:ieu-b-102 | rs4713692 | T | C | 0.367808 | -0.0276128 | 0.00498634 | 3.06507e-08 | 30.6659990869914 |
| Gastroesophageal reflux disease \|\| id:ebi-a-GCST90000514 | Major depression \|\| id:ieu-b-102 | rs569356 | G | A | 0.140835 | -0.037919 | 0.00690971 | 4.07005e-08 | 30.1157796119383 |
| Gastroesophageal reflux disease \|\| id:ebi-a-GCST90000514 | Major depression \|\| id:ieu-b-102 | rs6711584 | A | G | 0.452019 | 0.0322545 | 0.00483974 | 2.65583e-11 | 44.4157055825809 |
| Gastroesophageal reflux disease \|\| id:ebi-a-GCST90000514 | Major depression \|\| id:ieu-b-102 | rs6722661 | A | G | 0.364669 | -0.0322543 | 0.00500354 | 1.14623e-10 | 41.554732408618 |
| Gastroesophageal reflux disease \|\| id:ebi-a-GCST90000514 | Major depression \|\| id:ieu-b-102 | rs6780459 | T | A | 0.746622 | 0.0305507 | 0.0055211 | 3.13993e-08 | 30.6190071747621 |
| Gastroesophageal reflux disease \|\| id:ebi-a-GCST90000514 | Major depression \|\| id:ieu-b-102 | rs7032155 | A | C | 0.59185 | 0.02775 | 0.00491358 | 1.62686e-08 | 31.8955365412296 |
| Gastroesophageal reflux disease \|\| id:ebi-a-GCST90000514 | Major depression \|\| id:ieu-b-102 | rs7206608 | G | C | 0.322927 | 0.0291541 | 0.005145 | 1.45761e-08 | 32.109128634422 |
| Gastroesophageal reflux disease \|\| id:ebi-a-GCST90000514 | Major depression \|\| id:ieu-b-102 | rs7241572 | A | G | 0.209101 | 0.0365511 | 0.00597464 | 9.49336e-10 | 37.4263451829351 |
| Gastroesophageal reflux disease \|\| id:ebi-a-GCST90000514 | Major depression \|\| id:ieu-b-102 | rs7527682 | G | A | 0.53725 | -0.026684 | 0.00482174 | 3.12831e-08 | 30.626282702619 |
| Gastroesophageal reflux disease \|\| id:ebi-a-GCST90000514 | Major depression \|\| id:ieu-b-102 | rs7541875 | G | A | 0.426069 | 0.0273972 | 0.00484976 | 1.61213e-08 | 31.9133110932094 |
| Gastroesophageal reflux disease \|\| id:ebi-a-GCST90000514 | Major depression \|\| id:ieu-b-102 | rs7600261 | T | C | 0.306391 | 0.0338034 | 0.00522051 | 9.47327e-11 | 41.9271077920917 |
| Gastroesophageal reflux disease \|\| id:ebi-a-GCST90000514 | Major depression \|\| id:ieu-b-102 | rs7612999 | A | G | 0.245338 | 0.0305231 | 0.00559546 | 4.89824e-08 | 29.7567631431059 |
| Gastroesophageal reflux disease \|\| id:ebi-a-GCST90000514 | Major depression \|\| id:ieu-b-102 | rs761777 | G | A | 0.254034 | 0.0345341 | 0.00554469 | 4.71455e-10 | 38.7919618879507 |
| Gastroesophageal reflux disease \|\| id:ebi-a-GCST90000514 | Major depression \|\| id:ieu-b-102 | rs7675588 | A | C | 0.794635 | -0.0335228 | 0.00595438 | 1.80264e-08 | 31.696220362102 |
| Gastroesophageal reflux disease \|\| id:ebi-a-GCST90000514 | Major depression \|\| id:ieu-b-102 | rs7685686 | G | A | 0.422353 | -0.0279222 | 0.00489191 | 1.14427e-08 | 32.5793451155023 |
| Gastroesophageal reflux disease \|\| id:ebi-a-GCST90000514 | Major depression \|\| id:ieu-b-102 | rs773109 | A | G | 0.335269 | -0.0380572 | 0.00510213 | 8.71365e-14 | 55.6378866086894 |
| Gastroesophageal reflux disease \|\| id:ebi-a-GCST90000514 | Major depression \|\| id:ieu-b-102 | rs7942368 | T | C | 0.214659 | -0.0339683 | 0.00591917 | 9.54135e-09 | 32.9325984247732 |
| Gastroesophageal reflux disease \|\| id:ebi-a-GCST90000514 | Major depression \|\| id:ieu-b-102 | rs903959 | A | T | 0.399262 | 0.0291631 | 0.00491608 | 2.9893e-09 | 35.1908284437607 |
| Gastroesophageal reflux disease \|\| id:ebi-a-GCST90000514 | Major depression \|\| id:ieu-b-102 | rs9373363 | G | A | 0.253631 | -0.0326836 | 0.00555966 | 4.13457e-09 | 34.5591702770283 |
| Gastroesophageal reflux disease \|\| id:ebi-a-GCST90000514 | Major depression \|\| id:ieu-b-102 | rs9396740 | A | G | 0.248794 | -0.031493 | 0.0055587 | 1.46562e-08 | 32.0982676657298 |
| Gastroesophageal reflux disease \|\| id:ebi-a-GCST90000514 | Major depression \|\| id:ieu-b-102 | rs942065 | A | G | 0.634045 | 0.0307384 | 0.00500925 | 8.44559e-10 | 37.6545187902854 |
| Gastroesophageal reflux disease \|\| id:ebi-a-GCST90000514 | Major depression \|\| id:ieu-b-102 | rs9517313 | C | G | 0.383217 | 0.0331144 | 0.00494054 | 2.04786e-11 | 44.9246747256703 |
| Gastroesophageal reflux disease \|\| id:ebi-a-GCST90000514 | Major depression \|\| id:ieu-b-102 | rs9529055 | A | G | 0.475633 | 0.0266604 | 0.00481637 | 3.10549e-08 | 30.6403440066952 |
| Gastroesophageal reflux disease \|\| id:ebi-a-GCST90000514 | Major depression \|\| id:ieu-b-102 | rs9542729 | G | C | 0.20244 | -0.0363194 | 0.00599914 | 1.41205e-09 | 36.6521399815746 |
| Gastroesophageal reflux disease \|\| id:ebi-a-GCST90000514 | Major depression \|\| id:ieu-b-102 | rs9615905 | T | C | 0.458193 | 0.0275657 | 0.0048378 | 1.21233e-08 | 32.467005186298 |
| Gastroesophageal reflux disease \|\| id:ebi-a-GCST90000514 | Major depression \|\| id:ieu-b-102 | rs9636202 | A | G | 0.26663 | -0.035044 | 0.00547191 | 1.51022e-10 | 41.0156361689272 |
| Gastroesophageal reflux disease \|\| id:ebi-a-GCST90000514 | Major depression \|\| id:ieu-b-102 | rs9940128 | A | G | 0.421755 | 0.0332513 | 0.00486305 | 8.05749e-12 | 46.7519563532776 |

SNPs, single nucleotide polymorphisms; EAF, Effect allele frequency.
