# Supplementary material for: Mendelian randomization study of gastroesophageal reflux disease and major depression
Source: PLoS One. 2023 Sep 28;18(9):e0291086. doi: 10.1371/journal.pone.0291086 (PMC10538746; doi:10.1371/journal.pone.0291086)
Supplement: S3 Table — SNPs, single nucleotide polymorphisms. (DOCX) [file pone.0291086.s005.docx]

**Table S3. Leave-one-out results of exposure with the risk of outcome.**

| Exposure | Outcome | Sample Size | SNP | b | se | *P* |
| --- | --- | --- | --- | --- | --- | --- |
| Major Depressive Disorder \|\| id:ieu-a-1187 | Gastroesophageal reflux disease \|\| id:ebi-a-GCST90000514 | 602604 | rs10149470 | 0.41698492 | 0.06678802 | *4.2814E-10* |
| Major Depressive Disorder \|\| id:ieu-a-1187 | Gastroesophageal reflux disease \|\| id:ebi-a-GCST90000514 | 602604 | rs10950398 | 0.38501722 | 0.06371471 | *1.5143E-09* |
| Major Depressive Disorder \|\| id:ieu-a-1187 | Gastroesophageal reflux disease \|\| id:ebi-a-GCST90000514 | 602604 | rs10959913 | 0.39190366 | 0.06647306 | *3.7315E-09* |
| Major Depressive Disorder \|\| id:ieu-a-1187 | Gastroesophageal reflux disease \|\| id:ebi-a-GCST90000514 | 602604 | rs11135349 | 0.41411805 | 0.06734322 | *7.7794E-10* |
| Major Depressive Disorder \|\| id:ieu-a-1187 | Gastroesophageal reflux disease \|\| id:ebi-a-GCST90000514 | 602604 | rs11643192 | 0.41752243 | 0.06615352 | *2.7649E-10* |
| Major Depressive Disorder \|\| id:ieu-a-1187 | Gastroesophageal reflux disease \|\| id:ebi-a-GCST90000514 | 602604 | rs11663393 | 0.38651682 | 0.06447617 | *2.0383E-09* |
| Major Depressive Disorder \|\| id:ieu-a-1187 | Gastroesophageal reflux disease \|\| id:ebi-a-GCST90000514 | 602604 | rs11682175 | 0.42395293 | 0.06464135 | *5.4337E-11* |
| Major Depressive Disorder \|\| id:ieu-a-1187 | Gastroesophageal reflux disease \|\| id:ebi-a-GCST90000514 | 602604 | rs1226412 | 0.41444899 | 0.0669114 | *5.8658E-10* |
| Major Depressive Disorder \|\| id:ieu-a-1187 | Gastroesophageal reflux disease \|\| id:ebi-a-GCST90000514 | 602604 | rs12552 | 0.41581309 | 0.06917811 | *1.8465E-09* |
| Major Depressive Disorder \|\| id:ieu-a-1187 | Gastroesophageal reflux disease \|\| id:ebi-a-GCST90000514 | 602604 | rs12958048 | 0.39256511 | 0.06696705 | *4.5715E-09* |
| Major Depressive Disorder \|\| id:ieu-a-1187 | Gastroesophageal reflux disease \|\| id:ebi-a-GCST90000514 | 602604 | rs1354115 | 0.40301773 | 0.06768598 | *2.613E-09* |
| Major Depressive Disorder \|\| id:ieu-a-1187 | Gastroesophageal reflux disease \|\| id:ebi-a-GCST90000514 | 602604 | rs1432639 | 0.39719492 | 0.06871333 | *7.4495E-09* |
| Major Depressive Disorder \|\| id:ieu-a-1187 | Gastroesophageal reflux disease \|\| id:ebi-a-GCST90000514 | 602604 | rs159963 | 0.40283109 | 0.06766605 | *2.6291E-09* |
| Major Depressive Disorder \|\| id:ieu-a-1187 | Gastroesophageal reflux disease \|\| id:ebi-a-GCST90000514 | 602604 | rs2005864 | 0.40685948 | 0.06779481 | *1.957E-09* |
| Major Depressive Disorder \|\| id:ieu-a-1187 | Gastroesophageal reflux disease \|\| id:ebi-a-GCST90000514 | 602604 | rs2389016 | 0.3875957 | 0.06491615 | *2.3622E-09* |
| Major Depressive Disorder \|\| id:ieu-a-1187 | Gastroesophageal reflux disease \|\| id:ebi-a-GCST90000514 | 602604 | rs247910 | 0.42742513 | 0.06458755 | *3.6468E-11* |
| Major Depressive Disorder \|\| id:ieu-a-1187 | Gastroesophageal reflux disease \|\| id:ebi-a-GCST90000514 | 602604 | rs4074723 | 0.41544637 | 0.06668869 | *4.6752E-10* |
| Major Depressive Disorder \|\| id:ieu-a-1187 | Gastroesophageal reflux disease \|\| id:ebi-a-GCST90000514 | 602604 | rs4904738 | 0.39167018 | 0.06626526 | *3.4078E-09* |
| Major Depressive Disorder \|\| id:ieu-a-1187 | Gastroesophageal reflux disease \|\| id:ebi-a-GCST90000514 | 602604 | rs6905391 | 0.3931808 | 0.06737595 | *5.3589E-09* |
| Major Depressive Disorder \|\| id:ieu-a-1187 | Gastroesophageal reflux disease \|\| id:ebi-a-GCST90000514 | 602604 | rs7430565 | 0.41580607 | 0.06691857 | *5.178E-10* |
| Major Depressive Disorder \|\| id:ieu-a-1187 | Gastroesophageal reflux disease \|\| id:ebi-a-GCST90000514 | 602604 | rs7856424 | 0.39707659 | 0.06718883 | *3.424E-09* |
| Major Depressive Disorder \|\| id:ieu-a-1187 | Gastroesophageal reflux disease \|\| id:ebi-a-GCST90000514 | 602604 | rs8025231 | 0.3895891 | 0.06694286 | *5.8944E-09* |
| Major Depressive Disorder \|\| id:ieu-a-1187 | Gastroesophageal reflux disease \|\| id:ebi-a-GCST90000514 | 602604 | rs915057 | 0.42266175 | 0.0655251 | *1.1157E-10* |
| Major Depressive Disorder \|\| id:ieu-a-1187 | Gastroesophageal reflux disease \|\| id:ebi-a-GCST90000514 | 602604 | rs9427672 | 0.41786315 | 0.06600943 | *2.446E-10* |
| Major Depressive Disorder \|\| id:ieu-a-1187 | Gastroesophageal reflux disease \|\| id:ebi-a-GCST90000514 | 602604 | All | 0.40529861 | 0.06513905 | *4.9069E-10* |
| Major depression \|\| id:ieu-b-102 | Gastroesophageal reflux disease \|\| id:ebi-a-GCST90000514 | 602604 | rs1021363 | 0.71183943 | 0.04991842 | *3.8815E-46* |
| Major depression \|\| id:ieu-b-102 | Gastroesophageal reflux disease \|\| id:ebi-a-GCST90000514 | 602604 | rs10235664 | 0.70811602 | 0.04780926 | *1.2387E-49* |
| Major depression \|\| id:ieu-b-102 | Gastroesophageal reflux disease \|\| id:ebi-a-GCST90000514 | 602604 | rs10913112 | 0.72499077 | 0.05051402 | *1.0311E-46* |
| Major depression \|\| id:ieu-b-102 | Gastroesophageal reflux disease \|\| id:ebi-a-GCST90000514 | 602604 | rs12919291 | 0.73115499 | 0.04944898 | *1.8023E-49* |
| Major depression \|\| id:ieu-b-102 | Gastroesophageal reflux disease \|\| id:ebi-a-GCST90000514 | 602604 | rs12967143 | 0.72525403 | 0.05087443 | *4.1274E-46* |
| Major depression \|\| id:ieu-b-102 | Gastroesophageal reflux disease \|\| id:ebi-a-GCST90000514 | 602604 | rs13037326 | 0.71951659 | 0.05069799 | *1.0238E-45* |
| Major depression \|\| id:ieu-b-102 | Gastroesophageal reflux disease \|\| id:ebi-a-GCST90000514 | 602604 | rs150346963 | 0.72814893 | 0.05033779 | *2.0086E-47* |
| Major depression \|\| id:ieu-b-102 | Gastroesophageal reflux disease \|\| id:ebi-a-GCST90000514 | 602604 | rs17641524 | 0.73661114 | 0.04756535 | *4.2931E-54* |
| Major depression \|\| id:ieu-b-102 | Gastroesophageal reflux disease \|\| id:ebi-a-GCST90000514 | 602604 | rs1931388 | 0.72356394 | 0.05079861 | *4.9007E-46* |
| Major depression \|\| id:ieu-b-102 | Gastroesophageal reflux disease \|\| id:ebi-a-GCST90000514 | 602604 | rs1950829 | 0.72240814 | 0.05087158 | *9.0802E-46* |
| Major depression \|\| id:ieu-b-102 | Gastroesophageal reflux disease \|\| id:ebi-a-GCST90000514 | 602604 | rs2111592 | 0.72374742 | 0.05054954 | *1.6992E-46* |
| Major depression \|\| id:ieu-b-102 | Gastroesophageal reflux disease \|\| id:ebi-a-GCST90000514 | 602604 | rs2214123 | 0.71972912 | 0.05058627 | *6.1628E-46* |
| Major depression \|\| id:ieu-b-102 | Gastroesophageal reflux disease \|\| id:ebi-a-GCST90000514 | 602604 | rs2232423 | 0.71583105 | 0.05139456 | *4.2729E-44* |
| Major depression \|\| id:ieu-b-102 | Gastroesophageal reflux disease \|\| id:ebi-a-GCST90000514 | 602604 | rs2418449 | 0.72110101 | 0.05064722 | *5.3442E-46* |
| Major depression \|\| id:ieu-b-102 | Gastroesophageal reflux disease \|\| id:ebi-a-GCST90000514 | 602604 | rs2522831 | 0.71925286 | 0.05051813 | *5.3595E-46* |
| Major depression \|\| id:ieu-b-102 | Gastroesophageal reflux disease \|\| id:ebi-a-GCST90000514 | 602604 | rs2568958 | 0.73100312 | 0.0508891 | *8.6264E-47* |
| Major depression \|\| id:ieu-b-102 | Gastroesophageal reflux disease \|\| id:ebi-a-GCST90000514 | 602604 | rs30266 | 0.71536851 | 0.05090488 | *7.3792E-45* |
| Major depression \|\| id:ieu-b-102 | Gastroesophageal reflux disease \|\| id:ebi-a-GCST90000514 | 602604 | rs354155 | 0.73064255 | 0.04966777 | *5.5119E-49* |
| Major depression \|\| id:ieu-b-102 | Gastroesophageal reflux disease \|\| id:ebi-a-GCST90000514 | 602604 | rs3807865 | 0.71685143 | 0.0507387 | *2.5421E-45* |
| Major depression \|\| id:ieu-b-102 | Gastroesophageal reflux disease \|\| id:ebi-a-GCST90000514 | 602604 | rs4141983 | 0.72925823 | 0.04986393 | *1.9467E-48* |
| Major depression \|\| id:ieu-b-102 | Gastroesophageal reflux disease \|\| id:ebi-a-GCST90000514 | 602604 | rs4497414 | 0.71921112 | 0.05075645 | *1.4081E-45* |
| Major depression \|\| id:ieu-b-102 | Gastroesophageal reflux disease \|\| id:ebi-a-GCST90000514 | 602604 | rs4799949 | 0.70770306 | 0.04873672 | *8.9276E-48* |
| Major depression \|\| id:ieu-b-102 | Gastroesophageal reflux disease \|\| id:ebi-a-GCST90000514 | 602604 | rs4936276 | 0.71718651 | 0.05052824 | *1.0012E-45* |
| Major depression \|\| id:ieu-b-102 | Gastroesophageal reflux disease \|\| id:ebi-a-GCST90000514 | 602604 | rs59283172 | 0.71854152 | 0.05048488 | *5.7334E-46* |
| Major depression \|\| id:ieu-b-102 | Gastroesophageal reflux disease \|\| id:ebi-a-GCST90000514 | 602604 | rs61914045 | 0.7288718 | 0.05009909 | *5.9608E-48* |
| Major depression \|\| id:ieu-b-102 | Gastroesophageal reflux disease \|\| id:ebi-a-GCST90000514 | 602604 | rs62535714 | 0.72768139 | 0.05017103 | *1.1427E-47* |
| Major depression \|\| id:ieu-b-102 | Gastroesophageal reflux disease \|\| id:ebi-a-GCST90000514 | 602604 | rs66511648 | 0.72184335 | 0.05071643 | *5.7232E-46* |
| Major depression \|\| id:ieu-b-102 | Gastroesophageal reflux disease \|\| id:ebi-a-GCST90000514 | 602604 | rs7152906 | 0.71737117 | 0.05046715 | *7.4358E-46* |
| Major depression \|\| id:ieu-b-102 | Gastroesophageal reflux disease \|\| id:ebi-a-GCST90000514 | 602604 | rs7241572 | 0.71138118 | 0.04946369 | *6.7236E-47* |
| Major depression \|\| id:ieu-b-102 | Gastroesophageal reflux disease \|\| id:ebi-a-GCST90000514 | 602604 | rs72948506 | 0.70965876 | 0.0487023 | *4.2729E-48* |
| Major depression \|\| id:ieu-b-102 | Gastroesophageal reflux disease \|\| id:ebi-a-GCST90000514 | 602604 | rs7538938 | 0.72696691 | 0.05026793 | *2.1107E-47* |
| Major depression \|\| id:ieu-b-102 | Gastroesophageal reflux disease \|\| id:ebi-a-GCST90000514 | 602604 | rs754287 | 0.73847296 | 0.04780673 | *7.8977E-54* |
| Major depression \|\| id:ieu-b-102 | Gastroesophageal reflux disease \|\| id:ebi-a-GCST90000514 | 602604 | rs7725715 | 0.73776639 | 0.04832179 | *1.2537E-52* |
| Major depression \|\| id:ieu-b-102 | Gastroesophageal reflux disease \|\| id:ebi-a-GCST90000514 | 602604 | rs9364755 | 0.7183066 | 0.05043328 | *4.9759E-46* |
| Major depression \|\| id:ieu-b-102 | Gastroesophageal reflux disease \|\| id:ebi-a-GCST90000514 | 602604 | rs9529218 | 0.71245671 | 0.04986535 | *2.6142E-46* |
| Major depression \|\| id:ieu-b-102 | Gastroesophageal reflux disease \|\| id:ebi-a-GCST90000514 | 602604 | rs9536381 | 0.71802928 | 0.05041721 | *5.0449E-46* |
| Major depression \|\| id:ieu-b-102 | Gastroesophageal reflux disease \|\| id:ebi-a-GCST90000514 | 602604 | rs9831648 | 0.71290041 | 0.04978555 | *1.6549E-46* |
| Major depression \|\| id:ieu-b-102 | Gastroesophageal reflux disease \|\| id:ebi-a-GCST90000514 | 602604 | All | 0.72158653 | 0.04936762 | *2.2014E-48* |
| Gastroesophageal reflux disease \|\| id:ebi-a-GCST90000514 | Major Depressive Disorder \|\| id:ieu-a-1187 | 480359 | rs1021363 | 0.65055612 | 0.08424976 | *1.1474E-14* |
| Gastroesophageal reflux disease \|\| id:ebi-a-GCST90000514 | Major Depressive Disorder \|\| id:ieu-a-1187 | 480359 | rs12204714 | 0.65964548 | 0.08557887 | *1.2776E-14* |
| Gastroesophageal reflux disease \|\| id:ebi-a-GCST90000514 | Major Depressive Disorder \|\| id:ieu-a-1187 | 480359 | rs12967855 | 0.69192951 | 0.09456299 | *2.5333E-13* |
| Gastroesophageal reflux disease \|\| id:ebi-a-GCST90000514 | Major Depressive Disorder \|\| id:ieu-a-1187 | 480359 | rs2043539 | 0.6478323 | 0.07831987 | *1.3215E-16* |
| Gastroesophageal reflux disease \|\| id:ebi-a-GCST90000514 | Major Depressive Disorder \|\| id:ieu-a-1187 | 480359 | rs3793577 | 0.67438213 | 0.09058119 | *9.6903E-14* |
| Gastroesophageal reflux disease \|\| id:ebi-a-GCST90000514 | Major Depressive Disorder \|\| id:ieu-a-1187 | 480359 | rs6711584 | 0.73577339 | 0.07596401 | *3.4643E-22* |
| Gastroesophageal reflux disease \|\| id:ebi-a-GCST90000514 | Major Depressive Disorder \|\| id:ieu-a-1187 | 480359 | rs9396740 | 0.69386275 | 0.090818 | *2.1698E-14* |
| Gastroesophageal reflux disease \|\| id:ebi-a-GCST90000514 | Major Depressive Disorder \|\| id:ieu-a-1187 | 480359 | rs9940128 | 0.72417279 | 0.08391254 | *6.1304E-18* |
| Gastroesophageal reflux disease \|\| id:ebi-a-GCST90000514 | Major Depressive Disorder \|\| id:ieu-a-1187 | 480359 | All | 0.68420725 | 0.08017248 | *1.4114E-17* |
| Gastroesophageal reflux disease \|\| id:ebi-a-GCST90000514 | Major depression \|\| id:ieu-b-102 | 500199 | rs10010963 | 0.47510486 | 0.02705066 | *4.6875E-69* |
| Gastroesophageal reflux disease \|\| id:ebi-a-GCST90000514 | Major depression \|\| id:ieu-b-102 | 500199 | rs1011407 | 0.47873482 | 0.02721076 | *2.7585E-69* |
| Gastroesophageal reflux disease \|\| id:ebi-a-GCST90000514 | Major depression \|\| id:ieu-b-102 | 500199 | rs10133111 | 0.47657313 | 0.02725323 | *1.8046E-68* |
| Gastroesophageal reflux disease \|\| id:ebi-a-GCST90000514 | Major depression \|\| id:ieu-b-102 | 500199 | rs1021363 | 0.47094866 | 0.02637462 | *2.5899E-71* |
| Gastroesophageal reflux disease \|\| id:ebi-a-GCST90000514 | Major depression \|\| id:ieu-b-102 | 500199 | rs10837002 | 0.47815184 | 0.02721722 | *4.3326E-69* |
| Gastroesophageal reflux disease \|\| id:ebi-a-GCST90000514 | Major depression \|\| id:ieu-b-102 | 500199 | rs11762636 | 0.47563306 | 0.02737495 | *1.2815E-67* |
| Gastroesophageal reflux disease \|\| id:ebi-a-GCST90000514 | Major depression \|\| id:ieu-b-102 | 500199 | rs11953061 | 0.47623031 | 0.027173 | *9.0946E-69* |
| Gastroesophageal reflux disease \|\| id:ebi-a-GCST90000514 | Major depression \|\| id:ieu-b-102 | 500199 | rs12204714 | 0.47603367 | 0.02716864 | *9.8304E-69* |
| Gastroesophageal reflux disease \|\| id:ebi-a-GCST90000514 | Major depression \|\| id:ieu-b-102 | 500199 | rs12357321 | 0.48112852 | 0.02702328 | *6.5538E-71* |
| Gastroesophageal reflux disease \|\| id:ebi-a-GCST90000514 | Major depression \|\| id:ieu-b-102 | 500199 | rs12453010 | 0.48142019 | 0.02698203 | *3.3205E-71* |
| Gastroesophageal reflux disease \|\| id:ebi-a-GCST90000514 | Major depression \|\| id:ieu-b-102 | 500199 | rs12598916 | 0.4752552 | 0.02714392 | *1.2294E-68* |
| Gastroesophageal reflux disease \|\| id:ebi-a-GCST90000514 | Major depression \|\| id:ieu-b-102 | 500199 | rs12967855 | 0.47169242 | 0.02679013 | *2.1787E-69* |
| Gastroesophageal reflux disease \|\| id:ebi-a-GCST90000514 | Major depression \|\| id:ieu-b-102 | 500199 | rs12997558 | 0.47918824 | 0.02717366 | *1.3437E-69* |
| Gastroesophageal reflux disease \|\| id:ebi-a-GCST90000514 | Major depression \|\| id:ieu-b-102 | 500199 | rs13107325 | 0.48047433 | 0.02725235 | *1.4353E-69* |
| Gastroesophageal reflux disease \|\| id:ebi-a-GCST90000514 | Major depression \|\| id:ieu-b-102 | 500199 | rs1334297 | 0.48094691 | 0.02717246 | *4.2085E-70* |
| Gastroesophageal reflux disease \|\| id:ebi-a-GCST90000514 | Major depression \|\| id:ieu-b-102 | 500199 | rs13409451 | 0.47818322 | 0.02722437 | *4.605E-69* |
| Gastroesophageal reflux disease \|\| id:ebi-a-GCST90000514 | Major depression \|\| id:ieu-b-102 | 500199 | rs1431196 | 0.47296313 | 0.02692895 | *4.7E-69* |
| Gastroesophageal reflux disease \|\| id:ebi-a-GCST90000514 | Major depression \|\| id:ieu-b-102 | 500199 | rs1479405 | 0.48150895 | 0.02698181 | *3.1224E-71* |
| Gastroesophageal reflux disease \|\| id:ebi-a-GCST90000514 | Major depression \|\| id:ieu-b-102 | 500199 | rs1510719 | 0.47767714 | 0.02738259 | *3.7865E-68* |
| Gastroesophageal reflux disease \|\| id:ebi-a-GCST90000514 | Major depression \|\| id:ieu-b-102 | 500199 | rs1596747 | 0.4781201 | 0.02727651 | *8.6622E-69* |
| Gastroesophageal reflux disease \|\| id:ebi-a-GCST90000514 | Major depression \|\| id:ieu-b-102 | 500199 | rs1716171 | 0.47851754 | 0.02727812 | *6.8266E-69* |
| Gastroesophageal reflux disease \|\| id:ebi-a-GCST90000514 | Major depression \|\| id:ieu-b-102 | 500199 | rs17379561 | 0.48072421 | 0.02724078 | *1.0687E-69* |
| Gastroesophageal reflux disease \|\| id:ebi-a-GCST90000514 | Major depression \|\| id:ieu-b-102 | 500199 | rs1883842 | 0.47807442 | 0.02723274 | *5.4335E-69* |
| Gastroesophageal reflux disease \|\| id:ebi-a-GCST90000514 | Major depression \|\| id:ieu-b-102 | 500199 | rs1937450 | 0.47804501 | 0.02728417 | *9.9126E-69* |
| Gastroesophageal reflux disease \|\| id:ebi-a-GCST90000514 | Major depression \|\| id:ieu-b-102 | 500199 | rs2016933 | 0.47697264 | 0.02722395 | *1.0024E-68* |
| Gastroesophageal reflux disease \|\| id:ebi-a-GCST90000514 | Major depression \|\| id:ieu-b-102 | 500199 | rs2023878 | 0.48186102 | 0.02688871 | *8.149E-72* |
| Gastroesophageal reflux disease \|\| id:ebi-a-GCST90000514 | Major depression \|\| id:ieu-b-102 | 500199 | rs2106353 | 0.47908495 | 0.0272372 | *2.9721E-69* |
| Gastroesophageal reflux disease \|\| id:ebi-a-GCST90000514 | Major depression \|\| id:ieu-b-102 | 500199 | rs215614 | 0.48038428 | 0.02717965 | *6.6027E-70* |
| Gastroesophageal reflux disease \|\| id:ebi-a-GCST90000514 | Major depression \|\| id:ieu-b-102 | 500199 | rs2164300 | 0.48002477 | 0.02709726 | *3.2189E-70* |
| Gastroesophageal reflux disease \|\| id:ebi-a-GCST90000514 | Major depression \|\| id:ieu-b-102 | 500199 | rs2240326 | 0.48494045 | 0.02716587 | *2.8343E-71* |
| Gastroesophageal reflux disease \|\| id:ebi-a-GCST90000514 | Major depression \|\| id:ieu-b-102 | 500199 | rs2396133 | 0.47372368 | 0.02693835 | *3.1832E-69* |
| Gastroesophageal reflux disease \|\| id:ebi-a-GCST90000514 | Major depression \|\| id:ieu-b-102 | 500199 | rs2396766 | 0.481266 | 0.02709349 | *1.3633E-70* |
| Gastroesophageal reflux disease \|\| id:ebi-a-GCST90000514 | Major depression \|\| id:ieu-b-102 | 500199 | rs2734839 | 0.47310988 | 0.02676017 | *6.0189E-70* |
| Gastroesophageal reflux disease \|\| id:ebi-a-GCST90000514 | Major depression \|\| id:ieu-b-102 | 500199 | rs2744961 | 0.48345452 | 0.02652639 | *3.244E-74* |
| Gastroesophageal reflux disease \|\| id:ebi-a-GCST90000514 | Major depression \|\| id:ieu-b-102 | 500199 | rs2782641 | 0.47972517 | 0.02712789 | *5.5864E-70* |
| Gastroesophageal reflux disease \|\| id:ebi-a-GCST90000514 | Major depression \|\| id:ieu-b-102 | 500199 | rs2834005 | 0.47840105 | 0.02722749 | *4.1439E-69* |
| Gastroesophageal reflux disease \|\| id:ebi-a-GCST90000514 | Major depression \|\| id:ieu-b-102 | 500199 | rs2838771 | 0.47779598 | 0.02721308 | *5.2044E-69* |
| Gastroesophageal reflux disease \|\| id:ebi-a-GCST90000514 | Major depression \|\| id:ieu-b-102 | 500199 | rs324769 | 0.47411411 | 0.02691301 | *1.8392E-69* |
| Gastroesophageal reflux disease \|\| id:ebi-a-GCST90000514 | Major depression \|\| id:ieu-b-102 | 500199 | rs329122 | 0.47837554 | 0.02723494 | *4.5851E-69* |
| Gastroesophageal reflux disease \|\| id:ebi-a-GCST90000514 | Major depression \|\| id:ieu-b-102 | 500199 | rs3766823 | 0.48203784 | 0.026908 | *9.1222E-72* |
| Gastroesophageal reflux disease \|\| id:ebi-a-GCST90000514 | Major depression \|\| id:ieu-b-102 | 500199 | rs3793577 | 0.47327809 | 0.02676383 | *5.6198E-70* |
| Gastroesophageal reflux disease \|\| id:ebi-a-GCST90000514 | Major depression \|\| id:ieu-b-102 | 500199 | rs3828917 | 0.47437838 | 0.02693187 | *1.9231E-69* |
| Gastroesophageal reflux disease \|\| id:ebi-a-GCST90000514 | Major depression \|\| id:ieu-b-102 | 500199 | rs3863241 | 0.47735113 | 0.02729577 | *1.7655E-68* |
| Gastroesophageal reflux disease \|\| id:ebi-a-GCST90000514 | Major depression \|\| id:ieu-b-102 | 500199 | rs4300861 | 0.47393252 | 0.02699169 | *5.1245E-69* |
| Gastroesophageal reflux disease \|\| id:ebi-a-GCST90000514 | Major depression \|\| id:ieu-b-102 | 500199 | rs4382592 | 0.48049381 | 0.02707032 | *1.7284E-70* |
| Gastroesophageal reflux disease \|\| id:ebi-a-GCST90000514 | Major depression \|\| id:ieu-b-102 | 500199 | rs4713692 | 0.47552047 | 0.02710033 | *6.3075E-69* |
| Gastroesophageal reflux disease \|\| id:ebi-a-GCST90000514 | Major depression \|\| id:ieu-b-102 | 500199 | rs569356 | 0.47493622 | 0.02702645 | *3.9652E-69* |
| Gastroesophageal reflux disease \|\| id:ebi-a-GCST90000514 | Major depression \|\| id:ieu-b-102 | 500199 | rs6711584 | 0.47668103 | 0.02727645 | *2.1859E-68* |
| Gastroesophageal reflux disease \|\| id:ebi-a-GCST90000514 | Major depression \|\| id:ieu-b-102 | 500199 | rs6722661 | 0.4835827 | 0.02666724 | *1.7191E-73* |
| Gastroesophageal reflux disease \|\| id:ebi-a-GCST90000514 | Major depression \|\| id:ieu-b-102 | 500199 | rs6780459 | 0.47945551 | 0.02715741 | *9.3653E-70* |
| Gastroesophageal reflux disease \|\| id:ebi-a-GCST90000514 | Major depression \|\| id:ieu-b-102 | 500199 | rs7032155 | 0.47505599 | 0.02706267 | *5.551E-69* |
| Gastroesophageal reflux disease \|\| id:ebi-a-GCST90000514 | Major depression \|\| id:ieu-b-102 | 500199 | rs7206608 | 0.47981163 | 0.0271343 | *5.6851E-70* |
| Gastroesophageal reflux disease \|\| id:ebi-a-GCST90000514 | Major depression \|\| id:ieu-b-102 | 500199 | rs7241572 | 0.47231451 | 0.02666068 | *3.1684E-70* |
| Gastroesophageal reflux disease \|\| id:ebi-a-GCST90000514 | Major depression \|\| id:ieu-b-102 | 500199 | rs7527682 | 0.47802286 | 0.02722236 | *4.9933E-69* |
| Gastroesophageal reflux disease \|\| id:ebi-a-GCST90000514 | Major depression \|\| id:ieu-b-102 | 500199 | rs7541875 | 0.47752908 | 0.02723131 | *7.6059E-69* |
| Gastroesophageal reflux disease \|\| id:ebi-a-GCST90000514 | Major depression \|\| id:ieu-b-102 | 500199 | rs7600261 | 0.47351625 | 0.02696026 | *4.6902E-69* |
| Gastroesophageal reflux disease \|\| id:ebi-a-GCST90000514 | Major depression \|\| id:ieu-b-102 | 500199 | rs7612999 | 0.4810895 | 0.02694551 | *2.6827E-71* |
| Gastroesophageal reflux disease \|\| id:ebi-a-GCST90000514 | Major depression \|\| id:ieu-b-102 | 500199 | rs761777 | 0.47631376 | 0.0272124 | *1.3459E-68* |
| Gastroesophageal reflux disease \|\| id:ebi-a-GCST90000514 | Major depression \|\| id:ieu-b-102 | 500199 | rs7675588 | 0.47792951 | 0.02722933 | *5.7412E-69* |
| Gastroesophageal reflux disease \|\| id:ebi-a-GCST90000514 | Major depression \|\| id:ieu-b-102 | 500199 | rs7685686 | 0.47370461 | 0.02683459 | *9.6902E-70* |
| Gastroesophageal reflux disease \|\| id:ebi-a-GCST90000514 | Major depression \|\| id:ieu-b-102 | 500199 | rs773109 | 0.4849895 | 0.02664116 | *4.752E-74* |
| Gastroesophageal reflux disease \|\| id:ebi-a-GCST90000514 | Major depression \|\| id:ieu-b-102 | 500199 | rs7942368 | 0.47977169 | 0.02714368 | *6.5034E-70* |
| Gastroesophageal reflux disease \|\| id:ebi-a-GCST90000514 | Major depression \|\| id:ieu-b-102 | 500199 | rs903959 | 0.48330835 | 0.02659866 | *8.855E-74* |
| Gastroesophageal reflux disease \|\| id:ebi-a-GCST90000514 | Major depression \|\| id:ieu-b-102 | 500199 | rs9373363 | 0.47496894 | 0.02707581 | *6.8246E-69* |
| Gastroesophageal reflux disease \|\| id:ebi-a-GCST90000514 | Major depression \|\| id:ieu-b-102 | 500199 | rs9396740 | 0.47481972 | 0.02704554 | *5.3231E-69* |
| Gastroesophageal reflux disease \|\| id:ebi-a-GCST90000514 | Major depression \|\| id:ieu-b-102 | 500199 | rs942065 | 0.47809847 | 0.02725417 | *6.8214E-69* |
| Gastroesophageal reflux disease \|\| id:ebi-a-GCST90000514 | Major depression \|\| id:ieu-b-102 | 500199 | rs9517313 | 0.47500215 | 0.02717515 | *2.0608E-68* |
| Gastroesophageal reflux disease \|\| id:ebi-a-GCST90000514 | Major depression \|\| id:ieu-b-102 | 500199 | rs9529055 | 0.47591542 | 0.02714408 | *8.0309E-69* |
| Gastroesophageal reflux disease \|\| id:ebi-a-GCST90000514 | Major depression \|\| id:ieu-b-102 | 500199 | rs9542729 | 0.4722368 | 0.02663316 | *2.4102E-70* |
| Gastroesophageal reflux disease \|\| id:ebi-a-GCST90000514 | Major depression \|\| id:ieu-b-102 | 500199 | rs9615905 | 0.47881721 | 0.02720065 | *2.3298E-69* |
| Gastroesophageal reflux disease \|\| id:ebi-a-GCST90000514 | Major depression \|\| id:ieu-b-102 | 500199 | rs9636202 | 0.47731628 | 0.02726287 | *1.2466E-68* |
| Gastroesophageal reflux disease \|\| id:ebi-a-GCST90000514 | Major depression \|\| id:ieu-b-102 | 500199 | rs9940128 | 0.4814894 | 0.02709611 | *1.2137E-70* |
| Gastroesophageal reflux disease \|\| id:ebi-a-GCST90000514 | Major depression \|\| id:ieu-b-102 | 500199 | All | 0.47775002 | 0.0268812 | *1.1513E-70* |

SNPs, single nucleotide polymorphisms.
